# An Expanded View on the Morphological Diversity of Long-Nosed Antlion Larvae Further Supports a Decline of Silky Lacewings in the Past 100 Million Years

**DOI:** 10.3390/insects14020170

**Published:** 2023-02-09

**Authors:** Colin Hassenbach, Laura Buchner, Gideon T. Haug, Carolin Haug, Joachim T. Haug

**Affiliations:** 1 Faculty of Biology, Biocenter, Ludwig-Maximilians-Universität München (LMU Munich), Großhaderner Str. 2, 82152 Planegg-Martinsried, Germany; 2 GeoBio-Center at LMU, Richard-Wagner-Str. 10, 80333 Munich, Germany

**Keywords:** Psychopsidae, Neuroptera, Myanmar amber, Burmese amber, Cretaceous, quantitative morphology

## Abstract

**Simple Summary:**

The insect group Neuroptera (lacewings) is often claimed to have been more diverse in the past than today. The same appears to have also been the case not only for the entire group Neuroptera, but also for several of its ingroups. Silky lacewings (Psychopsidae) are represented by relatively few species today. Their larvae, also called long-nosed antlions, can easily be identified by the following characteristics: they resemble antlion larvae, lack teeth in their stylets (mouthparts for catching prey), have trumpet-shaped attachment structures on their legs, and have prominent forward-directed upper lips (labra, singular labrum). Therefore, these larvae can also be recognised in the fossil record. An earlier study demonstrated a decline in the morphological diversity of long-nosed antlion larvae over the past 100 million years. Here, we report several dozen new fossil long-nosed antlion larvae. With these, we expand the earlier quantitative analysis. Moreover, in this study, we can show that the morphological diversity of long-nosed antlion larvae has decreased over the past 100 million years. However, we apparently do not have the full original morphological diversity of long-nosed antlions available, as there is no sign of visible saturation yet.

**Abstract:**

Lacewings have been suggested to be a relict group. This means that the group of lacewings, Neuroptera, should have been more diverse in the past, which also applies to many ingroups of Neuroptera. Psychopsidae, the group of silky lacewings, is one of the ingroups of Neuroptera which is relatively species-poor in the modern fauna. Larvae of the group Psychopsidae, long-nosed antlions, can be easily identified as such in being larvae of antlion-like lacewings without teeth in their stylets (=compound structure of mandible and maxilla), with empodia (=attachment structures on legs) and with a prominent forward-protruding labrum. Therefore, such larvae can also be recognised in the fossil record. An earlier study demonstrated a decline in the morphological diversity of long-nosed antlion larvae over the past 100 million years. Here, we report several dozen new long-nosed antlion larvae and expand the earlier quantitative study. Our results further corroborate the decline of silky lacewings. Yet, a lack of an indication of saturation indicates that we have still not approached the original diversity of long-nosed antlions in the Cretaceous.

## 1. Introduction

The term “biodiversity” describes the overall variety of organisms around the world or in a certain habitat and plays an important role for our ecosystems. For years, people have seen a decline of biodiversity in different ecosystems [1,2,3,4,5,6,7,8,9,10], resulting in various efforts to protect species and their habitats. Most decline can be seen among representatives of the group Insecta, such as bees, beetles, or butterflies [2,5]. All of these play an important role in many ecological processes, for example, pollination or decomposition, and are in general essential for maintaining functional ecosystems [2,4,11,12,13,14,15,16].

The decline can be recognised on different aspects. Hallmann et al. [4] reported a 75% decline in biomass of flying representatives of Insecta over 27 years. Especially for longer time spans, reaching into the fossil record, biomass can only rarely be used for comparison. Another challenge when dealing with fossils is the inclusion of larvae, as these can also not easily be used in a taxonomic frame. Quantitative morphology offers a framework for overcoming both problems, facilitating a comparison of larval forms throughout the fossil history of a group. Such a comparative frame has been used for various lineages of lacewings [17,18,19,20,21,22,23,24,25,26,27].

Neuroptera, the group of lacewings, comprises approximately 6000 extant species [28,29,30,31]. The exact relationships within the group still seem not entirely settled [29,32,33,34,35,36,37,38,39,40,41,42,43].

The group Neuroptera is characterised by a specialisation of the larvae, the stylets, which are compound feeding structures formed by mandibles and maxillae [31,44,45,46,47,48,49,50]. Stylets come in various forms, including simple inward curved as, for example, in the groups Chrysopidae (green lacewings [51,52,53,54,55]), Hemerobiidae (brown lacewings [56,57,58,59,60]), some species of Mantispidae (mantis lacewings [47,61,62]), and many representatives of Mymeleontiformia (antlion-like lacewings [63,64,65,66,67]), though within the latter group, many of the stylets additionally bear teeth [47,50,68,69,70]. Stylets may be straight [71], as in Sisyridae [36,72,73,74,75], Berothidae [76,77,78,79,80], and many species of Mantispidae [35,77,81,82,83], or even outward-curved, as in Osmylidae [84,85,86,87,88]. The stylets allow the larvae to pierce prey, inject venom and saliva, and suck out the dissolved body tissues.

Most neuropterans undergo three larval stages before pupating [28,31] (see discussion in [22]). Larval stages can live for several years, while adults only live for up to a couple of weeks [28], indicating that most of their ecological impact is in fact generated by the larval forms.

Within Myrmeleontiformia, a now species-poor (28 species) but in the past more diverse group, are silky lacewings, Psychopsidae [89,90]. Modern representatives can be found in Australia, East and Southeast Asia, and Southern Africa [91,92]. Fossils are known from different regions around the world, suggesting that the modern distribution is only a relict one and that silky lacewings were much more diverse and widespread in the past [18,42,91,93]. The neuropteran lineage has been reconstructed to have emerged in the late Palaeozoic [42,94,95] and the group Psychopsidae in the late Triassic [91,96].

Larvae resembling the modern larvae of silky lacewings, also called long-nosed antlions, first appeared in the fossil record in Cretaceous ambers [18,90,97,98]. Modern long-nosed antlions live under leaf debris [99] or tree bark [28,99,100], making them predestined for becoming entrapped in amber. Long-nosed antlions can be easily recognised by their long protruding labrum (hence the name long-nosed antlions), in combination with their toothless stylets and trumpet-shaped empodia [18,101,102,103].

A previous quantitative morphology comparison of the head capsule of long-nosed antlions demonstrated a loss of shape diversity from the Cretaceous to the Eocene and further into the modern fauna [18], supporting earlier losses in diversity within Psychopsidae, as found in earlier reconstructions [91]. This loss also indicates a loss of ecological function. Here, we report new fossils of long-nosed antlions and expand the analysis of Haug et al. [18].

## 2. Material and Methods

### 2.1. Material

The study includes the specimens of Haug et al. [18]. One additional specimen in Eocene Baltic amber that had been overlooked by Haug et al. [18] was identified in the literature [104]. Images of another additional specimen from Eocene Baltic amber were kindly provided by Marius Veta (www.ambertreasure4u.com, accessed on 23 December 2022).

New material directly studied are specimens preserved in approximately 100 million years old Kachin amber, Myanmar [105,106,107,108]. Specimens were legally purchased via the trading platform ebay.com from various traders (burmitefossil, burmite-miner, burmite-researcher, cretaceous-burmite, macro-cretaceous). Specimens are deposited in the Palaeo-Evo-Devo Research Group Collection of Arthropods, Ludwig-Maximilians-University Munich, Germany, under repository numbers PED 0150, 0267, 0322, 0379, 0382, 0389, 0412, 0430, 0440, 0456, 0535, 0584, 0612, 0621, 0625, 0662, 0751, 0774, 0845, 0932, 0998, 1049, 1459, 1627, 1666, 1703, 1726, 1732, 1813, 1831, 1846, 1884, 1887, 1928, 1940, 1967, 2056, 2171, 2309, 2311, 2329, 2432, 2446, and 2448. This study adds 44 new Upper Cretaceous and 2 new Eocene long-nosed antlions to the growing list of fossil neuropteran larvae. In total, this makes 12 extant, 14 Eocene, and 72 Upper Cretaceous long-nosed antlions known so far. Information on the specimens is provided in Supplementary Materials File S1.

### 2.2. Imaging and Documentation

Directly studied specimens were imaged on a Keyence VHX 6000 digital microscope. Amber pieces were mounted on modelling clay in a Petri dish and covered by a drop of glycerol and a cover slip. The specimens were photographed using different lenses, providing magnification from 20-fold up to 2000-fold, under unpolarised ring light and cross-polarised coaxial light, on a white and black background, and in some cases with additional transmitted light. Besides “normal” imaging, the specimens were also documented under varying exposure times (HDR). All images are composite images from multiple smaller images with different focus layers stacked together as a panorama image to obtain an in-depth, high-resolution image. Specimens were photographed from the ventral and dorsal sides if accessible (see also details in [18]).

### 2.3. Image Processing and Presentation

The images were first processed using the built-in software of the Keyence VHX 6000 digital microscope. Additionally, colour saturation and sharpness were optimised using Adobe Photoshop CS2. Colour-marks of the different structures of the specimens were prepared to provide the reader with an interpretation of the accessible structures.

### 2.4. Measurements

Specimens were measured using the open-source software Fiji 2.0.0 (or ImageJ 1.53 [109]). If present, the total body length of the larvae (excluding the stylets), the length of the head capsule, and its width at the maximum expansion were measured.

### 2.5. Shape Analysis

Accessible head capsules and other traits were redrawn as vector graphics in Inkscape or Adobe Illustrator CS2. Shape analysis was conducted using the program SHAPE, following the method of Iwata and Ukai [110] and Braig et al. [111].

## 3. Results

### 3.1. Descriptions of New Fossil Larvae

**Figure 1:**
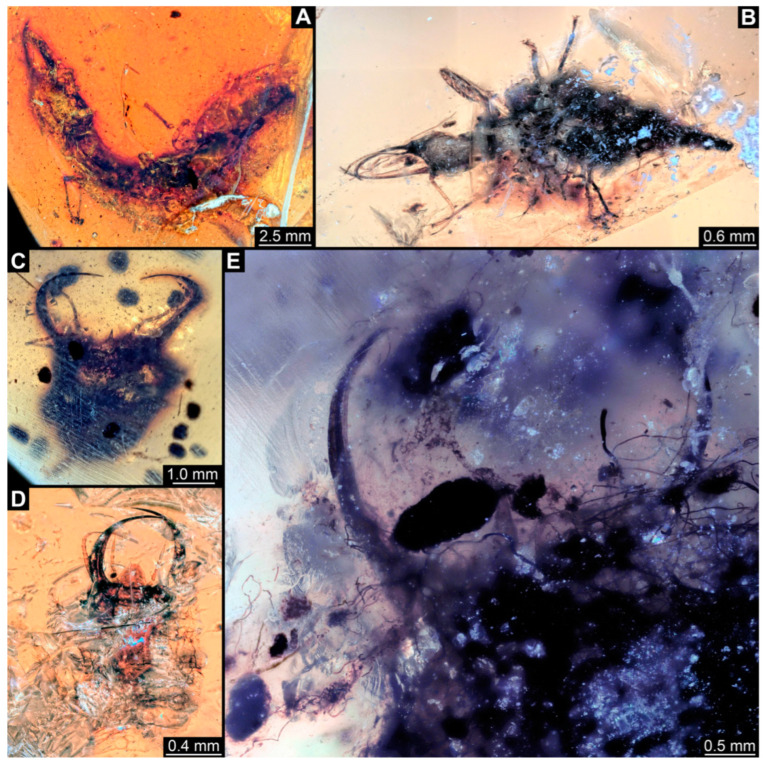
Specimens in Myanmar amber. (**A**) Specimen 71 (PED 0845), dorsal view. (**B**) Specimen 53 (PED 0150), dorsal view. (**C**) Specimen 67 (PED 0625). (**D**) Specimen 64 (PED 0584). (**E**) Specimen 54 (PED 0267).

**Figure 2:**
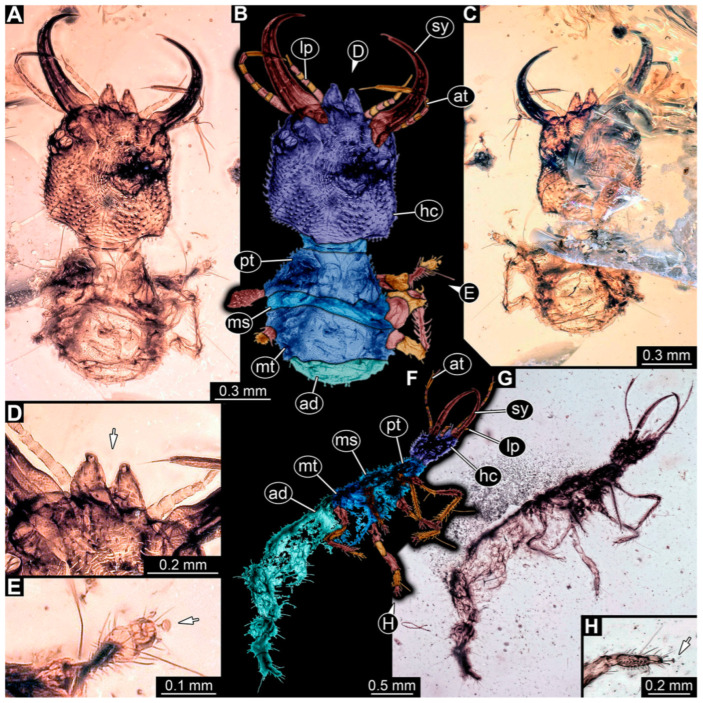
Specimens in Myanmar amber. (**A**–**E**) Specimen 62 (PED 0456). (**A**) Dorsal view. (**B**) Dorsal view, colour-marked. (**C**) Ventral view. (**D**) Close-up of labrum; arrow points to V-shaped split. (**E**) Close-up of first locomotory appendage; arrow marks empodium. (**F**–**H**) Specimen 55 (PED 0322). (**F**) Lateral view, colour-marked. (**G**) Lateral view. (**H**) Close-up of third locomotory appendage; arrow marks empodium. Abbreviations: ad = abdomen; at = antenna; hc = head capsule; lp = labial palp; ms = mesothorax; mt = metathorax; pt = prothorax; sy = stylet.

**Figure 3:**
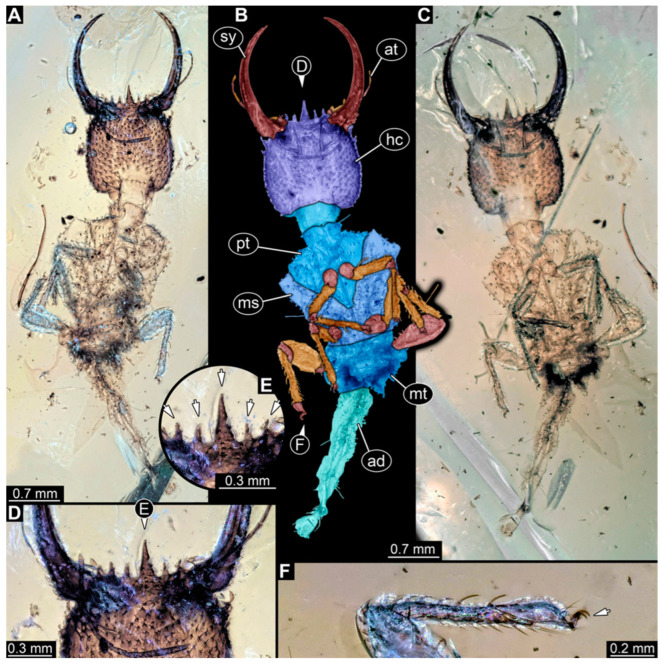
Specimen 56 (PED 0379); Myanmar amber. (**A**) Dorsal view. (**B**) Ventral view, colour-marked. (**C**) Ventral view. (**D**) Close-up of anterior head region. (**E**) Close-up of labrum in dorsal view; arrows mark spine-like protrusions. (**F**) Close-up of third locomotory appendage; arrow marks claw. Abbreviations: ad = abdomen; at = antenna; hc = head capsule; ms = mesothorax; mt = metathorax; pt = prothorax; sy = stylet.

**Figure 4:**
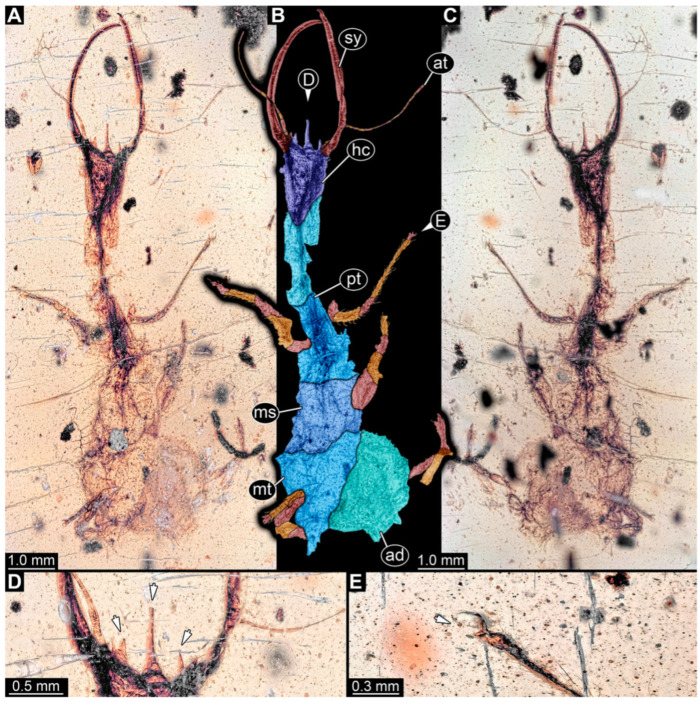
Specimen 57 (PED 0382); Myanmar amber. (**A**) Dorsal view. (**B**) Dorsal view, colour-marked. (**C**) Ventral view. (**D**) Close-up of labrum in dorsal view; arrows mark spine-like protrusions. (**E**) Close-up of first locomotory appendage; arrow marks empodium. Abbreviations: ad = abdomen; at = antenna; hc = head capsule; ms = mesothorax; mt = metathorax; pt = prothorax; sy = stylet.

**Figure 5:**
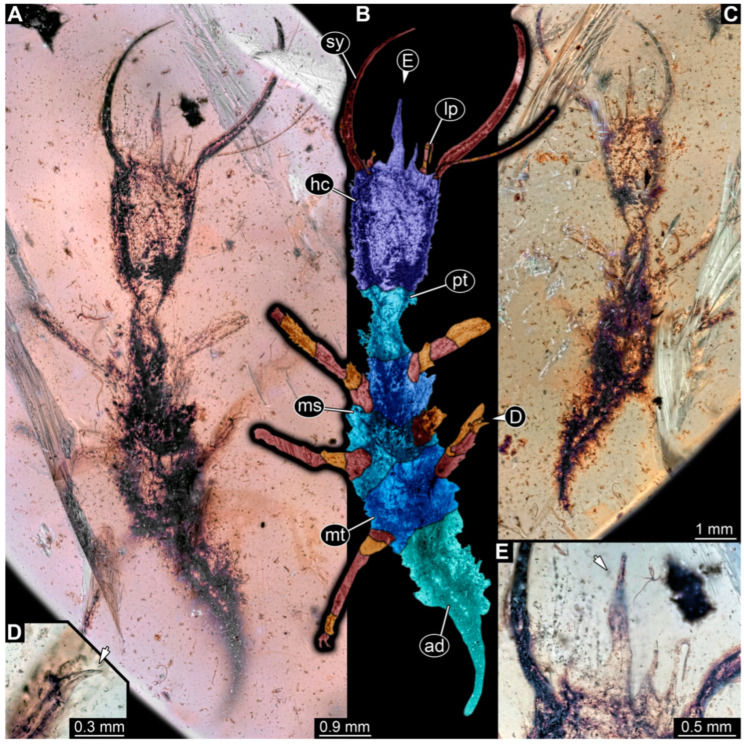
Specimen 58 (PED 0389); Myanmar amber. (**A**) Dorsal view. (**B**) Dorsal view, colour-marked. (**C**) Ventral view. (**D**) Close-up of third locomotory appendage; arrow marks claw. (**E**) Close-up of labrum in dorsal view; arrow marks middle spine-like protrusion. Abbreviations: ad = abdomen; hc = head capsule; lp = labial palp; ms = mesothorax; mt = metathorax; pt = prothorax; sy = stylet.

**Figure 6:**
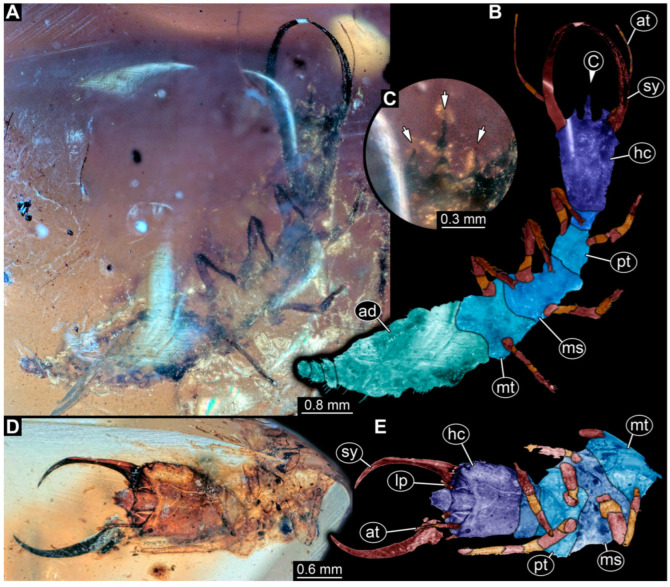
Specimens in Myanmar amber. (**A**–**C**) Specimen 65 (PED 0612). (**A**) Ventral view. (**B**) Ventral view, colour-marked. (**C**) Close-up of labrum in dorsal view; arrows mark spine-like protrusions. (**D**,**E**) Specimen 59 (PED 0412). (**D**) Ventral view. (**E**) Ventral view, colour-marked. Abbreviations: ad = abdomen; at = antenna; hc = head capsule; lp = labial palp; ms = mesothorax; mt = metathorax; pt = prothorax; sy = stylet.

**Figure 7:**
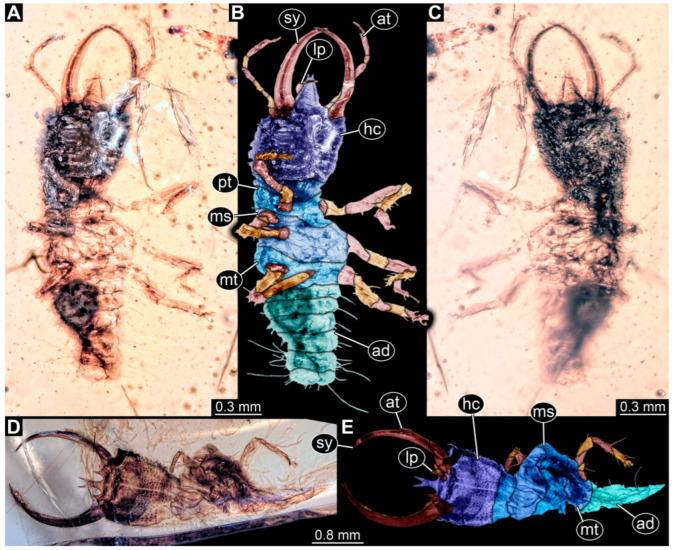
Specimens in Myanmar amber. (**A**–**C**) Specimen 63 (PED 0535). (**A**) Ventral view. (**B**) Ventral view, colour-marked. (**C**) Dorsal view. (**D**,**E**) Specimen 60 (PED 0430). (**D**) Dorsal view. (**E**) Dorsal view, colour-marked. Abbreviations: ad = abdomen; at = antenna; hc = head capsule; lp = labial palp; ms = mesothorax; mt = metathorax; pt = prothorax; sy = stylet.

**Figure 8:**
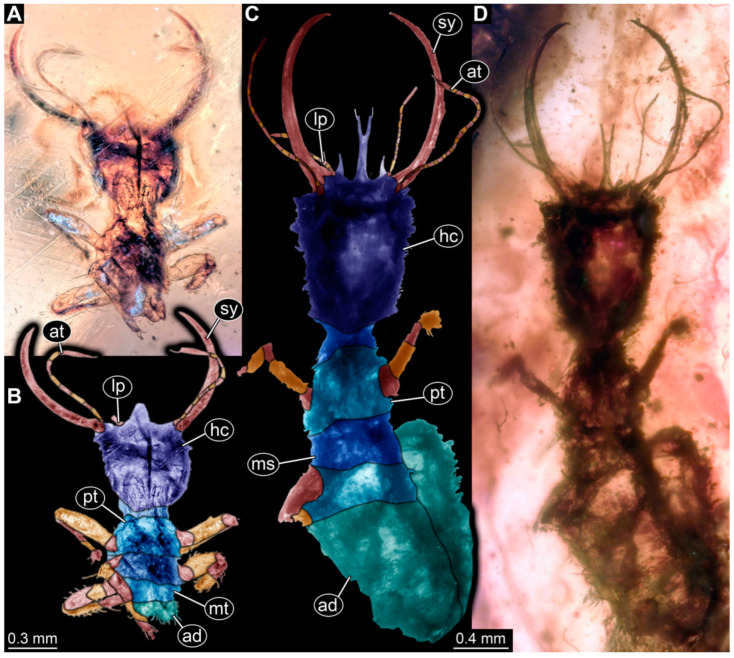
Specimens in Myanmar amber. (**A**,**B**) Specimen 61 (PED 0440). (**A**) Dorsal view. (**B**) Dorsal view, colour-marked. (**C**,**D**) Specimen 84 (PED 1884). (**C**) Dorsal view, colour-marked. (**D**) Dorsal view. Abbreviations: ad = abdomen; at = antenna; hc = head capsule; lp = labial palp; ms = mesothorax; mt = metathorax; pt = prothorax; sy = stylet.

(1) Specimen 53 (PED 0150) is preserved in Cretaceous Myanmar amber (Figure 1B). The dorsal and ventral sides are accessible. The head seems to be turned sideways or is deformed. The labrum is partly concealed; it therefore remains partly unclear whether this is definitely a long-nosed antlion. The antennae bear prominent setae at the distal ends. Trumpet-shaped empodia at the end of the locomotory appendages (legs) are visible. The abdomen is mostly covered by dirt or debris. The specimen has an approximate length of 3.6 mm. This specimen was not included in the final analysis.

(2) Specimen 54 (PED 0267) is preserved in Cretaceous Myanmar amber (Figure 1E). Only the head capsule is present, but it is largely covered by dirt or debris concealing the outer rim and labrum. The specimen has an estimated length of 14 mm. This specimen was not included in the final analysis.

(3) Specimen 55 (PED 0322) is preserved in Cretaceous Myanmar amber. Both lateral sides are accessible. The head is only accessible in the lateral view (Figure 2F,G). The labrum seems to have a triangular shape in the dorsal view. The antennae bear prominent setae at the distal ends. Trumpet-shaped empodia at the end of the locomotory appendages are visible (Figure 2H). The specimen has an approximate length of 3.9 mm. This specimen was not included in the final analysis.

(4) Specimen 56 (PED 0379) is preserved in Cretaceous Myanmar amber (Figure 3). The dorsal (Figure 3A) and ventral (Figure 3B,C) sides are accessible. The labrum is trident-like with a larger spine-like protrusion in the middle, but with two smaller spine-like protrusions next to the large spine on each side (Figure 3D,E); hence, it is not a trident in the strict sense, as it has five and not three prongs. No empodium is apparent, although the claws are well preserved (Figure 3F). The specimen has an approximate length of 5.5 mm.

(5) Specimen 57 (PED 0382) is preserved in Cretaceous Myanmar amber (Figure 4). The dorsal (Figure 4A,B) and ventral (Figure 4C) sides are accessible. The labrum is trident-like with a larger spine-like protrusion in the middle and two smaller spine-like protrusions next to the large spine (Figure 4D). An empodium is apparent (Figure 4E). The specimen has an approximate length of 8.6 mm.

(6) Specimen 58 (PED 0389) is preserved in Cretaceous Myanmar amber (Figure 5). The dorsal (Figure 5A,B) and ventral (Figure 5C) sides are accessible. The labrum is trident-like with a larger spine-like protrusion in the middle and two smaller spine-like protrusions next to the large spine (Figure 5E). The locomotory appendages are partly covered by a whitish coating (hereafter called Verlumung; adjective: verlumt) (Figure 5D). The specimen has an approximate length of 9.9 mm.

(7) Specimen 59 (PED 0412) is preserved in Cretaceous Myanmar amber (Figure 6D,E). The ventral side is accessible with parts of the abdomen being verlumt (Figure 6D,E). The dorsal side is largely concealed by dirt and partly by verlumt. One stylet is separated from the head capsule. The labrum is broad, pentagonal in the dorsal view, and bears small spine-like elevations at the corners. The specimen is located in a corner of the amber piece and the distal part of its abdomen appears to be missing. The specimen has an approximate length of 2.8 mm.

(8) Specimen 60 (PED 0430) is preserved in Cretaceous Myanmar amber. It is accessible from the dorsal side (Figure 7D,E). The labrum is trident-like with a large bifurcated middle spine-like protrusion and two smaller spine-like protrusions next to the large spine. The antennae bear prominent setae at the distal ends. Trumpet-shaped empodia at the end of the locomotory appendages are visible. Parts of the abdomen appear to be missing. The specimen has an approximate length of 3.6 mm.

(9) Specimen 61 (PED 0440) is preserved in Cretaceous Myanmar amber. It is accessible from the dorsal side (Figure 8A,B), but strongly verlumt from the ventral side. The labrum is triangular to pentagonal in the dorsal view. The antennae bear prominent setae at the distal ends. Trumpet-shaped empodia at the end of the locomotory appendages are visible. The specimen has an approximate length of 1.2 mm.

(10) Specimen 62 (PED 0456) is preserved in Cretaceous Myanmar amber (Figure 2A–E). The dorsal (Figure 2A,B) and ventral (Figure 2C) sides are accessible. The labrum is trapezoidal in the dorsal view with a large V-shaped split distally (Figure 2D). The antennae bear prominent setae at the distal ends. Trumpet-shaped empodia at the end of the locomotory appendages are visible (Figure 2E). The specimen has an approximate length of 1.5 mm.

(11) Specimen 63 (PED 0535) is preserved in Cretaceous Myanmar amber (Figure 7A–C). The dorsal (Figure 7C) and ventral (Figure 7A,B) sides are accessible. The labrum is triangular in the dorsal view. The antennae bear prominent setae at the distal ends. Trumpet-shaped empodia at the end of the locomotory appendages are visible. The abdomen is partly verlumt from the dorsal side. The specimen has an approximate length of 1.8 mm.

(12) Specimen 64 (PED 0584) is preserved in Cretaceous Myanmar amber (Figure 1D). The dorsal and ventral sides are strongly verlumt. Only the head and parts of the thorax are present. The labrum is triangular to pentagonal in the dorsal view. Trumpet-shaped empodia at the end of the locomotory appendages are visible. The specimen has an estimated length of 3.1–3.5 mm. This specimen was not included in the final analysis.

(13) Specimen 65 (PED 0612) is preserved in Cretaceous Myanmar amber (Figure 6A–C). It is accessible from the ventral side (Figure 6A,B), but strongly verlumt. The labrum is trident-like with a larger spine-like protrusion in the middle and two smaller spine-like protrusions next to the large spine (Figure 6C). The specimen has an approximate length of 6.9 mm.

(14) Specimen 66 (PED 0621) is preserved in Cretaceous Myanmar amber (Figure 9). The dorsal (Figure 9A,B) and ventral (Figure 9C) sides are accessible, but are partly concealed by dirt or Verlumung. The labrum is trident-like with a larger spine-like protrusion in the middle and two smaller spine-like protrusions next to the large spine (Figure 9D,E). The antennae bear prominent setae at the distal ends. The abdomen appears slim and elongated. The specimen has an approximate length of 5.7 mm.

(15) Specimen 67 (PED 0625) is preserved in Cretaceous Myanmar amber (Figure 1C). The dorsal and ventral sides are accessible, but strongly verlumt. Only the head is present, but a large area of the ventral side appears to be ground off near the neck region. The labrum appears to be trident-like with a larger spine-like protrusion in the middle and two smaller spine-like protrusions next to the large spine. The specimen has an estimated length of 12.4–15.8 mm. This specimen was not included in the final analysis.

(16) Specimen 68 (PED 0662) is preserved in Cretaceous Myanmar amber (Figure 10). The dorsal (Figure 10A) and ventral (Figure 10B,C) sides are accessible, but are partly concealed by dirt and Verlumung. The labrum is mostly concealed by Verlumung, but appears relatively broad and short (Figure 10D). The specimen has an approximate length of 8.3 mm.

(17) Specimen 69 (PED 0751) is preserved in Cretaceous Myanmar amber (Figure 11D–G). The ventral side is accessible, but partly verlumt (Figure 11D,E). The labrum is trident-like with a larger spine-like protrusion in the middle and two smaller spine-like protrusions next to the large spine (Figure 11F). Trumpet-shaped empodia at the end of the locomotory appendages are visible (Figure 11G). Large parts of the abdomen and thorax are concealed by a crack in the amber. The specimen has a measured length of approximately 5.0 mm, but an estimated length between 9.2–10.5 mm.

(18) Specimen 70 (PED 0774) is preserved in Cretaceous Myanmar amber (Figure 11A–C). The ventral side is accessible, but is strongly concealed by dirt and Verlumung (Figure 11A,B). The labrum is triangular in the dorsal view (Figure 11C). The antennae bear prominent setae at the distal ends. A part of the abdomen appears to be missing. The specimen has an estimated length of 2.5–2.7 mm.

(19) Specimen 71 (PED 0845) is preserved in Cretaceous Myanmar amber (Figure 1A). The dorsal and ventral sides are accessible, but are strongly concealed by dirt and Verlumung. The head seems to be turned sideways or experienced a deformation. Due to Verlumung, the shape of the labrum is not clearly discernible. The specimen has an approximate length of 15.7 mm. This specimen was not included in the final analysis.

(20) Specimen 72 (PED 0932) is preserved in Cretaceous Myanmar amber. The dorsal (Figure 12C) and ventral (Figure 12A,B) sides are accessible, but are partly concealed by dirt and Verlumung. The labrum is trident-like with a large bifurcated middle spine-like protrusion and two smaller spine-like protrusions next to the large spine (Figure 12D,E). The eyes are visible from the lateral view. The antennae bear prominent setae at the distal ends. The abdomen and thorax are mostly concealed. Trumpet-shaped empodia at the end of the locomotory appendages are visible. The specimen has an estimated length of 5.4–5.9 mm.

(21) Specimen 73 (PED 0998) is preserved in Cretaceous Myanmar amber (Figure 13). The dorsal (Figure 13A,B) and ventral (Figure 13C) sides are accessible, but are strongly concealed by dirt and Verlumung. The labrum is triangular with a shallow cleft distally. The abdomen appears slim and elongated. The specimen has an approximate length of 10.5 mm.

(22) Specimen 74 (PED 1049) is preserved in Cretaceous Myanmar amber. The dorsal and ventral sides are accessible, but are partly concealed by dirt and Verlumung. Only the head, neck, and parts of the prothorax are present (Figure 14A,B). The labrum is trident-like with a larger spine-like protrusion in the middle and two smaller spine-like protrusions next to the large spine. The specimen has an estimated length of 7.2–8.0 mm.

(23) Specimen 75 (PED 1459) is preserved in Cretaceous Myanmar amber (Figure 12F–I). The dorsal (Figure 12H) and ventral (Figure 12F,G) sides are accessible. The labrum is triangular in the dorsal view (Figure 12I). The antennae bear prominent setae at the distal ends. The specimen has an approximate length of 2.0 mm.

(24) Specimen 76 (PED 1627) is preserved in Cretaceous Myanmar amber (Figure 15). The dorsal (Figure 15A,B) and ventral (Figure 15C) sides are accessible, but are strongly concealed by dirt and Verlumung. The labrum appears relatively broad, short, and pentagonal in the dorsal view (Figure 15D,E). Trumpet-shaped empodia at the end of the locomotory appendages are visible (Figure 15F). The specimen has an approximate length of 10.1 mm.

(25) Specimen 77 (PED 1666) is preserved in Cretaceous Myanmar amber (Figure 16). The dorsal (Figure 16A,B) and ventral (Figure 16C) sides are accessible. The labrum is triangular to pentagonal in the dorsal view. The antennae bear prominent setae at the distal ends (Figure 16D). Trumpet-shaped empodia at the end of the locomotory appendages are visible (Figure 16F). Some setae have a peculiar fan-like shape (Figure 16E). The specimen has an approximate length of 1.9 mm.

(26) Specimen 78 (PED 1703) is preserved in Cretaceous Myanmar amber (Figure 17). The dorsal (Figure 17C) and ventral (Figure 17A,B) sides are accessible, but are strongly concealed by dirt and Verlumung. Half of one stylet is missing distally. The labrum appears pentagonal, but well rounded in the dorsal view (Figure 17D). Trumpet-shaped empodia at the end of the locomotory appendages are visible (Figure 17E). The specimen has an approximate length of 2.9 mm.

(27) Specimen 79 (PED 1726) is preserved in Cretaceous Myanmar amber (Figure 18). The dorsal (Figure 18C) and ventral (Figure 18A,B) sides are accessible, but are partly concealed by dirt and Verlumung. The labrum appears pentagonal in the dorsal view, and quite broad and short with a broad cleft distally (Figure 18D). The abdomen appears to be missing its distal end, which was presumably ground off. Trumpet-shaped empodia at the end of the locomotory appendages are visible (Figure 18E). The specimen has a measured length of approximately 7.6 mm, but an estimated length between 10.4–10.8 mm.

(28) Specimen 80 (PED 1732) is preserved in Cretaceous Myanmar amber (Figure 19). The dorsal (Figure 19A,B) and ventral (Figure 19C) sides are accessible. The stylets are partly concealed. The labrum is broad, pentagonal in the dorsal view, and bears small spine-like elevations at the corners (Figure 19D). No clear indications of an empodium are apparent (Figure 19E). The specimen has an approximate length of 7.7 mm.

(29) Specimen 81 (PED 1813) is preserved in Cretaceous Myanmar amber (Figure 20). The dorsal (Figure 20A,B) and ventral (Figure 20C) sides are accessible. The specimen appears to be the best preserved one among the new specimens. The labrum appears almost square-shaped in the dorsal view, with one bigger cleft in the middle distally and two flanking smaller clefts at the edges (Figure 20D). The antennae bear prominent setae at the distal ends. The thorax and abdomen are well-preserved and the subdivisions are easily recognisable. Trumpet-shaped empodia at the end of the locomotory appendages are visible (Figure 20E). The specimen has an approximate length of 3.5 mm.

(30) Specimen 82 (PED 1831) is preserved in Cretaceous Myanmar amber (Figure 14E,F). The ventral side is accessible. Only the head, neck, and parts of the prothorax are present. A small lateral part of the head capsule is missing. The labrum is triangular in the dorsal view. The specimen has an estimated length of 3.0–3.5 mm.

(31) Specimen 83 (PED 1846) is preserved in Cretaceous Myanmar amber (Figure 14C,D). The ventral side is accessible. Only the head, neck, and parts of the prothorax are present. The labrum appears almost square-shaped in the dorsal view, with one bigger cleft in the middle distally and two flanking smaller clefts at the edges. The antennae bear prominent setae at the distal ends. The specimen has an estimated length of 3.9–4.3 mm.

(32) Specimen 84 (PED 1884) is preserved in Cretaceous Myanmar amber (Figure 8C,D). The dorsal side is accessible, but is partly concealed by dirt and Verlumung. The labrum is trident-like with a large bifurcated middle spine-like protrusion and two smaller spine-like protrusions next to the large spine. The specimen has an approximate length of 3.4 mm.

(33) Specimen 85 (PED 1887) is preserved in Cretaceous Myanmar amber (Figure 21A–C). The ventral side is accessible, but is partly concealed by dirt and Verlumung (Figure 21A,B). One stylet is missing about one half distally. The labrum is broad, pentagonal in the dorsal view, and bears small spine-like elevations at the corners (Figure 21C). The antennae bear prominent setae at the distal ends (Figure 21C). The head capsule is separated from the body, with the whole neck region and parts of the adjacent head capsule missing. The body is missing the majority of the thorax and abdomen. Trumpet-shaped empodia at the end of the locomotory appendages are visible. The specimen has an estimated length of 4.5 mm. This specimen was not included in the final analysis.

(34) Specimen 86 (PED 1928) is preserved in Cretaceous Myanmar amber (Figure 22). The dorsal (Figure 22B,C) and ventral (Figure 22A) sides are accessible, but are partly concealed by dirt and Verlumung. The antennae bear prominent setae at the distal ends. The labrum is trident-like with a large bifurcated middle spine-like protrusion and two smaller spine-like protrusions next to the large spine (Figure 22D). The abdomen appears to be missing its distal end. Trumpet-shaped empodia at the end of the locomotory appendages are visible. The specimen has a measured length of approximately 4.0 mm, but an estimated length between 5.6–6.8 mm.

(35) Specimen 87 (PED 1940) is preserved in Cretaceous Myanmar amber (Figure 21D–G). The dorsal side is accessible, but is partly concealed by dirt and Verlumung (Figure 21D,E). The labrum is triangular in the dorsal view (Figure 21F,G). The antennae bear prominent setae at the distal ends. Parts of the abdomen seem to be concealed or missing. The specimen has a measured length of approximately 1.3 mm, but an estimated length between 2.3–2.8 mm.

(36) Specimen 88 (PED 1967) is preserved in Cretaceous Myanmar amber (Figure 23). The dorsal (Figure 23A,B) and ventral (Figure 23C) sides are accessible, but are largely concealed by dirt and Verlumung. The labrum is trident-like with a larger spine-like protrusion in the middle and two smaller spine-like protrusions next to the large spine (Figure 23D). The specimen has an approximate length of 11.9 mm.

(37) Specimen 89 (PED 2056) is preserved in Cretaceous amber from Myanmar (Figure 24). The specimen is well accessible in the dorsal view (Figure 24A,B), but is partly concealed by dirt in the ventral view (Figure 24C). The labrum is prominent, being triangular symmetric to trapezium-like in the dorsal view and relatively elongated. Trumpet-shaped empodia at the end of the locomotory appendages are visible (Figure 24D). The posterior trunk is missing. The specimen has an approximate length of 4.1 mm.

(38) Specimen 90 (PED 2171) is preserved in Cretaceous amber from Myanmar (Figure 25). The specimen is largely concealed by dirt and Verlumung in the ventral (Figure 25A,B) and dorsal views (Figure 25C). The labrum is prominent, being triangular symmetric to trapezium-like and relatively elongated. Each distal end of the walking appendages bears a trumpet-shaped empodium (Figure 25D). The specimen has an approximate length of 2.6 mm.

(39) Specimen 91 (PED 2309) is preserved in Cretaceous amber from Myanmar (Figure 26). The specimen is well accessible in the ventral (Figure 26A,B) and dorsal views (Figure 26C), but is partly concealed by Verlumung and dirt particles in the dorsal and ventral views. The labrum is prominent, being triangular symmetric to trapezium-like and relatively elongated (Figure 26D). The antennae bear prominent setae distally (Figure 26E). Each distal end of the walking appendages bears a trumpet-shaped empodium (Figure 26F). The specimen has an approximate length of 2.2 mm.

(40) Specimen 92 (PED 2311) is preserved in Cretaceous amber from Myanmar (Figure 27). The specimen is well accessible in the dorsal (Figure 27A,B) and ventral views (Figure 27C), but is partly concealed by Verlumung and dirt particles in the dorsal and ventral views. The labrum is prominent, being triangular symmetric to trapezium-like and very elongated. The specimen has an approximate length of 1.3 mm.

(41) Specimen 93 (PED 2329) is preserved in Cretaceous amber from Myanmar (Figure 28B). The trunk is missing completely, with only the head preserved. The head is well accessible in the dorsal view. The labrum is prominent, pentagonal, and relatively broad. The specimen has an approximate length of 1.8 mm.

(42) Specimen 94 (PED 2446) is preserved in Cretaceous amber from Myanmar (Figure 28A). The trunk is missing completely, with only the head preserved. The head is well accessible in the dorsal view. The labrum is prominent, triangular, and relatively elongated. The antennae bear prominent setae distally. The specimen has an approximate length of 2 mm.

(43) Specimen 95 (PED 2448) is preserved in Cretaceous amber from Myanmar (Figure 28C). The specimen is largely concealed by dirt particles in the dorsal and ventral views, with only the rough outline apparent. The specimen has an approximate size of 5 mm. This specimen was not included in the final analysis.

(44) Specimen 96 (PED 2432) is preserved in Cretaceous amber from Myanmar (Figure 29). The specimen is well accessible in the ventral (Figure 29A,B) and dorsal views (Figure 29C), but is partly concealed by Verlumung and dirt particles in the dorsal and ventral views. The labrum is prominent and simple triangular to pentagonal. The specimen has an approximate length of 8.5 mm.

(45) Specimen 97 is preserved in Baltic amber. It was configured by Ross [104] (his Figure 150, p. 87). Although the original photograph is relatively small and a large bubble conceals parts of the body, some general aspects can be well recognised (Figure 30A). The specimen was overlooked by Haug et al. [18]. The length was approximately 3 mm. The repository was not stated, but a similar-appearing specimen in an image repository was stated to show a specimen from the Natural History Museum, London.

(46) Specimen 98 is preserved in Baltic amber. The specimen was not directly investigated, but high-quality images (Figure 30B) were provided by Marius Veta (www.ambertreasure4u.com, accessed on 23 December 2022).

### 3.2. Shape Analysis

The results of the shape analyses are provided in Supplementary Materials File S2.

*Head and stylets*: The shape analysis of the head capsule with stylets resulted in six effective principal components (PCs), summarizing 94.2% of the overall References in the form of [XX] are not permitted in the images. Please move it to the figure caption. If necessary, please use the format of “Author+Year” instead in the images, and all mentioned references should be cited in the caption.of the dataset.

PC1 explains 52.5% of the overall variation. PC1 is dominated by the shape of the head and the mandibles. It describes very slender to very broad head shapes, with broad and long-to-narrow and short mandibles. Low values indicate a slender head with distally tapering mandibles, while high values indicate a broad head with mandibles that are in the distal region as broad as in the proximal region.

PC2 explains 24.8% of the overall variation. PC2 is dominated by the shape of the mandibles and the length of the head capsule, also focusing on the length of the labrum. Low values indicate a long head and labrum with relatively straight mandibles, while high values indicate a small head and small labrum with strongly curved mandible tips.

PC3 explains 7.5% of the overall variation. It is dominated by the length of the labrum. It describes a tapering-to-flattened labrum, yet the shape of the mandibles also influences this PC. Low values indicate an elongated tapering labrum with strongly curved mandible tips, while high values indicate a flattened, round labrum with straight mandible tips.

PC4 explains 3.9% of the overall variation. It is dominated by the length of the labrum. It describes long-to-short labra, yet the shape of the mandibles also seems to influence this PC. Low values indicate a long tapering labrum with mandibles that are in the distal region as broad as in the proximal region, while high values indicate a short tapering labrum with mandibles that are broad in the proximal region and tapering in the distal region.

PC5 explains 3.0% of the overall variation. It is dominated by the shape of the labrum and the shape of the mandibles. Low values indicate a short tapering labrum and mandibles that are broad in the proximal region and tapering in the distal region, while high values indicate a broad curved labrum with mandibles that are in the distal region as broad as in the proximal region.

PC6 explains 2.5% of the overall variation. It is dominated by the shape of the labrum, the mandibles, and the posterior head capsule. It describes a concave posterior rim and a tapering labrum with tapering mandibles to a convex posterior rim and a broad labrum with mandibles that are in the distal region as broad as in the proximal region.

*Head capsule*: The shape analysis of the head capsule resulted in eight effective principal components, summarizing 93.8% of the overall variation in the dataset.

PC1 explains 45.1% of the overall variation. PC1 is dominated by the shape of the head, especially focusing on the posterior rim. It describes a convex-to-concave posterior rim, yet the shape of the labrum also influences this PC. Low values indicate a convex posterior edge of the head with a tapering labrum, while high values indicate a concave posterior rim with a rounded labrum.

PC2 explains 18.6% of the overall variation. It is dominated by the shape of the head and describes rectangular-to-trapezium-like head capsules. Low values indicate a rectangular head with a round posterior and anterior rim, while high values indicate a round head with a tapered posterior and anterior rim.

PC3 explains 9.6% of the overall variation. It is dominated by the shape of the posterior rim and the length of the labrum. Low values indicate a posterior rim with an elongated labrum, while high values indicate a rectangular head with a short tapering labrum.

PC4 explains 7.5% of the overall variation. It is dominated by the shape of the posterior rim and the length of the labrum. Low values indicate a concave posterior rim with an elongated labrum, while high values indicate a round posterior rim with a short labrum.

PC5 explains 4.0% of the overall variation. It is dominated by the shape of the head capsule, yet the length of the labrum also seems to influence this PC. Low values indicate a trapezium-like head with a short labrum, while high values indicate a rectangular head with an elongated labrum.

PC6 explains 3.2% of the overall variation. It is dominated by the width of the head. It describes the lateral anterior rim of the head, yet the shape of the labrum also seems to influence this PC. Low values indicate a wide lateral anterior rim with a tapering labrum, while high values indicate a somewhat rectangular head with a broad labrum.

PC7 explains 2.7% of the overall variation. It is dominated by the shape of the labrum. Low values indicate an elongated tapering labrum, while high values indicate a wide-rounded labrum.

PC8 explains 2.2% of the overall variation. It is dominated by the shape of the anterior rim of the head. Low values indicate a tapering median anterior rim, while high values indicate a rectangular anterior rim.

*Stylets*: The shape analysis of the stylets resulted in four effective principal components, summarizing 96.5% of the overall variation in the dataset.

PC1 explains 79.6% of the overall variation. It is dominated by the curving of the mandibles. Low values indicate straight mandibles, while high values indicate rounded mandibles.

PC2 explains 10.9% of the overall variation. It is dominated by the curving of the mandibles and describes the curving of the anterior rim. Low values indicate curved mandibles with a rounded anterior rim, while high values indicate curved mandibles with a straight anterior rim.

PC3 explains 4.1% of the overall variation. It is dominated by the shape of the mandible tips. Low values indicate straight mandible tips, while high values indicate curved mandible tips.

PC4 explains 2.0% of the overall variation. It appears to be dominated by similar phenomena as PC3. Low values indicate curved mandible tips, while high values indicate straight mandible tips.

*Body outline with stylets*: The shape analysis of the entire body outline with stylets resulted in six effective principal components, summarizing 92.3% of the overall variation in the dataset.

PC1 explains 73.7% of the overall variation. It is dominated by the length of the mandibles in correlation with the length of the posterior rim of the body. Low values indicate long mandibles with a short sloping posterior rim, while high values indicate short mandibles with a long elongated posterior rim.

PC2 explains 14.4% of the overall variation. It is dominated by the length of the mandibles, yet the shape of the anterior rim also seems to influence this PC. Low values indicate long stout mandibles and a slightly curved anterior rim of the body, while high values indicate straight narrow mandibles and a straight anterior rim of the body.

PC3 explains 9.4% of the overall variation. It is dominated by the shape of the mandibles and describes narrow-to-broad mandibles. Low values indicate shortened narrow mandibles that are in the distal region as broad as in the proximal region, while high values indicate mandibles that are broad in the proximal region and tapering in the distal region.

PC4 explains 4.8% of the overall variation. It appears to be dominated by similar phenomena as PC3. Low values indicate curved mandibles, while high values indicate shorter straight mandibles.

PC5 explains 2.8% of the overall variation. It is dominated by the length of the labrum in correlation with the length of the mandibles. Low values indicate an elongated tapering labrum with elongated curved mandibles, while high values indicate a shorter round labrum with shorter mandibles.

PC6 explains 1.8% of the overall variation. It is dominated by the shape of the labrum. Low values indicate a rectangular labrum, while high values indicate a round labrum.

*Body outline without stylets*: The shape analysis of the entire body without stylets resulted in eight effective principal components, summarizing 92.9% of the overall variation in the dataset.

PC1 explains 39.5% of the overall variation. It is dominated by the shape of the thorax. Low values indicate a relatively concave thorax with a fluent transition between thorax and posterior trunk, while high values indicate a relatively convex thorax with a clear distinction from the posterior trunk.

PC2 explains 18.7% of the overall variation. It is dominated by the position of the narrowest part of the body, yet the length of the labrum also seems to influence this PC. Low values indicate a further anteriorly located narrowest part of the body and an elongated tapering labrum, while high values indicate a further posteriorly located narrowest part of the body and a shortened round labrum.

PC3 explains 12.3% of the overall variation. It is dominated by the shape of the head. Low values indicate a rectangular shape of the head, while high values indicate a tapering rim of the head.

PC4 explains 7.8% of the overall variation. It appears to be dominated by similar phenomena as PC3. Low values indicate a rectangular head, while high values indicate a round tapering head.

PC5 explains 5.3% of the overall variation. It is dominated by the shape of the labrum and describes round-to-tapering labra. Low values indicate a convex anterior rim with a round wide labrum, while high values indicate a concave anterior rim with a tapering labrum.

PC6 explains 4.3% of the overall variation. It is dominated by the position of the narrowest part of the body. Low values indicate a further anteriorly located narrowest part of the body, while high values indicate a further posteriorly located narrowest part of the body.

PC7 explains 3.1% of the overall variation. It is dominated by the position of the narrowest part of the body and the shape of the labrum. Low values indicate a further anteriorly located narrowest part of the body with a tapering labrum, while high values indicate a further posteriorly located narrowest part of the body with a shortened round labrum.

PC8 explains 2.0% of the overall variation. It seems to be dominated by the width of the head. Low values indicate a head as wide in the anterior region as in the posterior region, while high values indicate a head that is wide in the anterior region and narrow in the posterior region.

## 4. Discussion

### 4.1. General Observation: Loss of Diversity

This study of long-nosed antlions has expanded the analysis performed by Haug et al. [18], which had already found a decrease, or loss, of morphological diversity from the Cretaceous to the modern fauna. As we have only added new fossil forms, it is not surprising that this picture did not change. Still, we can recognise some differences to the earlier study. Moreover, more specimens may offer access to small details, such as the small fan-like setae observed in one of the specimens.

### 4.2. Head and Stylet Shape

**Figure 31:**
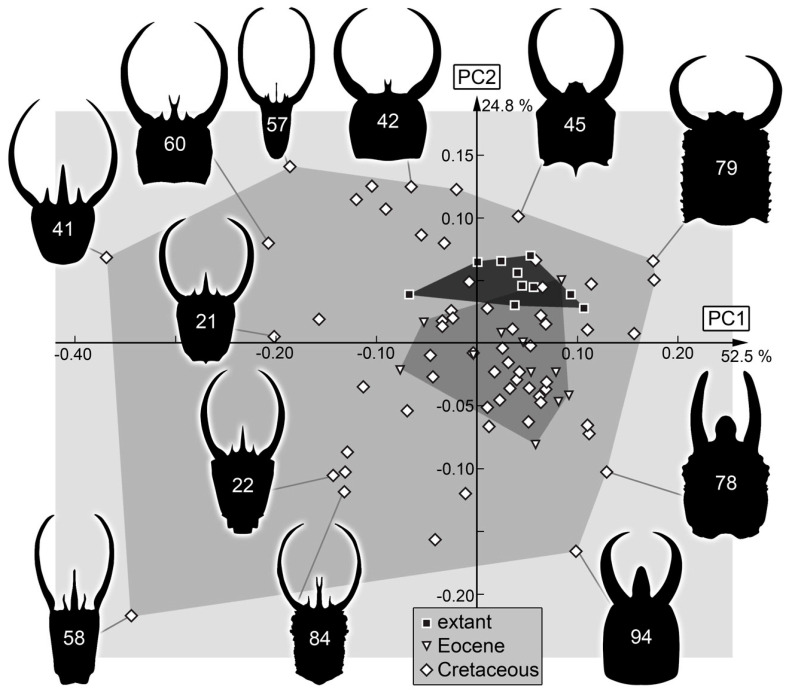
Scatterplot of PC2 vs. PC1 values of head + stylet shapes (see Supplementary Materials File S2 for details).

Although the original study concentrated on head capsule shape [18], most follow-up studies on larvae of other lineages of Neuroptera have used the head capsule together with the stylets [17,19,20,21,24,25,26,112]. This analysis (Figure 31) is also most comparable in its results to the earlier study. The morphospace occupation is the largest in the Cretaceous, is smaller in the Eocene, and even smaller in the modern fauna.

Yet, the relative sizes are different from the earlier study, being even larger in the Cretaceous. This should not be surprising, as many types of larvae that can be recognised on a qualitative level among the Cretaceous larvae are absent in the Eocene and modern fauna. Among these are many of the larvae with a trident-like labrum. Basically, the left half of the morphospace is occupied by trident-bearing larvae (Figure 31). Among these are also larvae with new morphologies: one with the middle spine of the trident being bifurcated but longer than in the already known larvae (specimen 84; Figure 8C) and one with more spines, i.e., five (specimen 56; Figure 3).

Moreover, other areas of the morphospace represent morphologies not seen in the modern fauna but now recognised by new specimens (Figure 31). In the upper right area of the morphospace, there are larvae with very broad labra (e.g., specimen 79; Figure 18). In the lower right of the morphospace, larvae plot which have relatively long labra in comparison to the rest of the head capsule, but are otherwise not special in their shape (e.g., specimen 94; Figure 28A).

**Figure 32:**
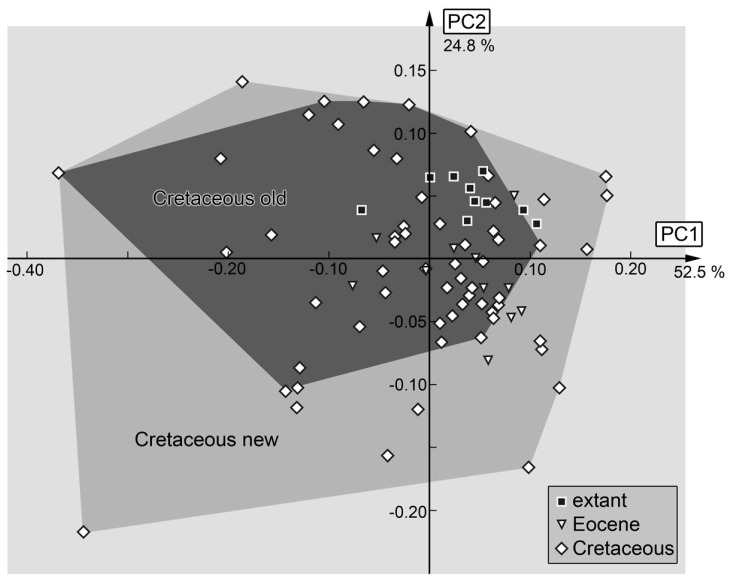
The same plot as in Figure 31, with area occupied by old specimens from Haug et al. [18] and combined area occupied by old and new specimens in comparison (see Supplementary Materials File S2 for details).

The influence of the new Cretaceous larvae is also seen when comparing the occupation of the morphospace of the larvae used in Haug et al. [18] and that of all of the Cretaceous larvae (Figure 32). It shows that the new specimens significantly increase the morphospace occupation. This implies that there is still no saturation effect, i.e., adding new specimens still increases the morphospace by adding new types of morphologies.

### 4.3. Stylet Shape

The stylet of long-nosed antlions is character-poor in comparison to other larvae of the group Myrmeleontiformia. In many other larvae, there are teeth that can vary in size, number, and position, but such teeth seem to have been secondarily lost in long-nosed antlions [113,114].

**Figure 33:**
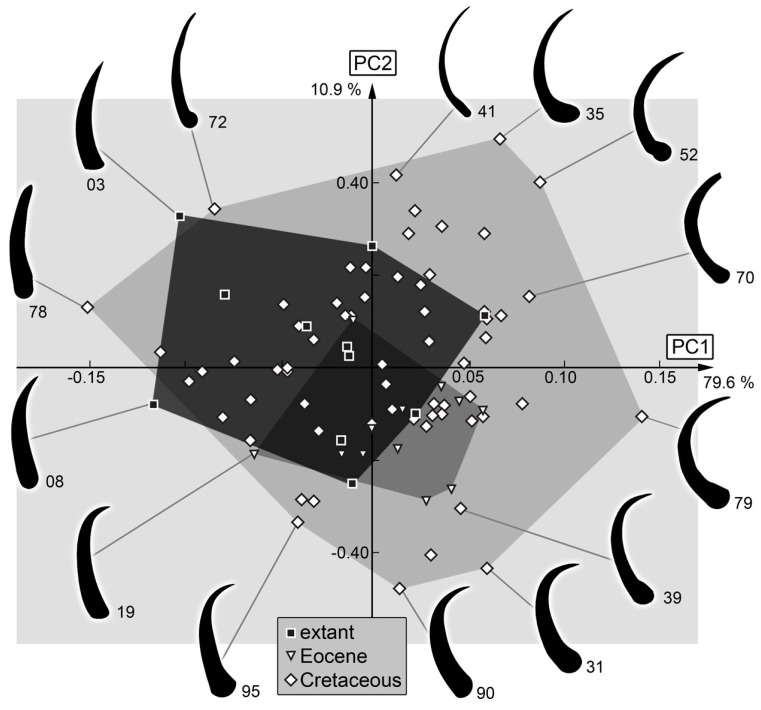
Scatterplot of PC2 vs. PC1 values of stylet shapes (see Supplementary Materials File S2 for details).

The main aspect that seems to vary within the stylet shape in long-nosed antlions is the curvature. In many fossils, the stylets seem stronger curved than in the modern larvae (Figure 33). A factor that seems to have not been strongly picked up by the shape analysis is the slenderness (or thickness) of the stylets. It appears that the trident-bearing larvae have slenderer stylets (Figure 31), but also vary strongly in curvature.

Similar to the plot of head and stylets, the Cretaceous larvae occupy the largest area of the morphospace, and this largely includes those of other groups. An important difference to plotting head together with stylets is that the occupation of the morphospace of the modern larvae is larger than that of the Eocene larvae. Yet, some Eocene larvae plot outside the range of the modern larvae, emphasising again that the Eocene fauna also differs from the modern fauna concerning insect larvae [115].

### 4.4. Head Capsule Shape

**Figure 34:**
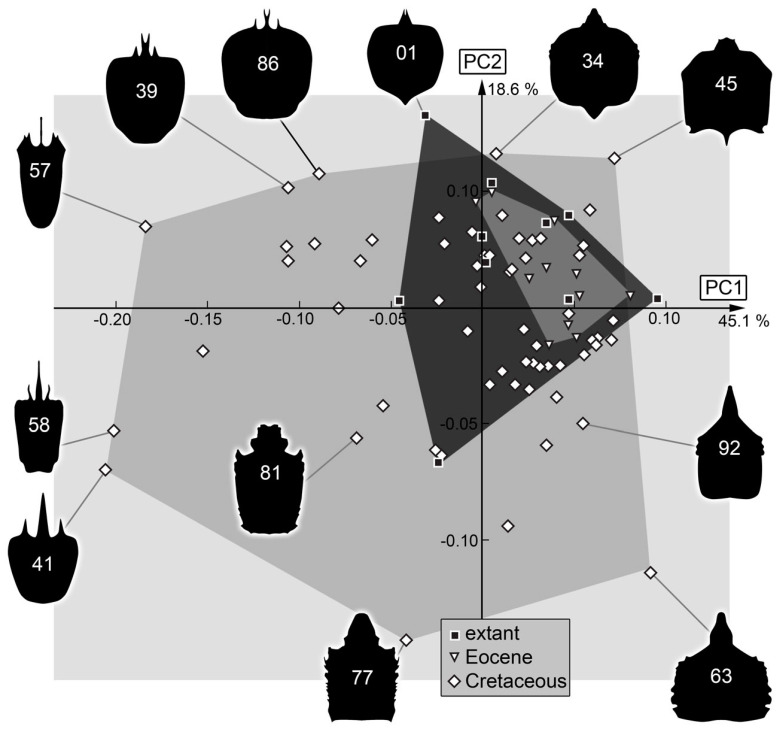
Scatterplot of PC2 vs. PC1 values of head capsule shapes (see Supplementary Materials File S2 for details).

Also in the head capsule shape, the Cretaceous larvae occupy the largest area, largely including the area of other groups (Figure 34). Similar to the stylets, the modern larvae occupy a larger area than the Eocene larvae. This is different to the earlier study by Haug et al. [18]. It is even more surprising as two new larvae from the Eocene were added to the dataset. It seems most likely that this difference to the earlier study is caused by the new Cretaceous larvae, as they have polarised the morphospace in a different way.

Another aspect to mention in this aspect is that, unlike in the stylets, the area occupied by Eocene larvae is entirely inside the area of the modern larvae. The fact that the combined analysis (of head and stylets) provides a different view than the two separate analyses for Eocene and modern larvae indicates that a major difference in the Eocene larvae is the relative size of the stylets in relation to the head capsule.

### 4.5. Body Shape including Stylets

**Figure 35:**
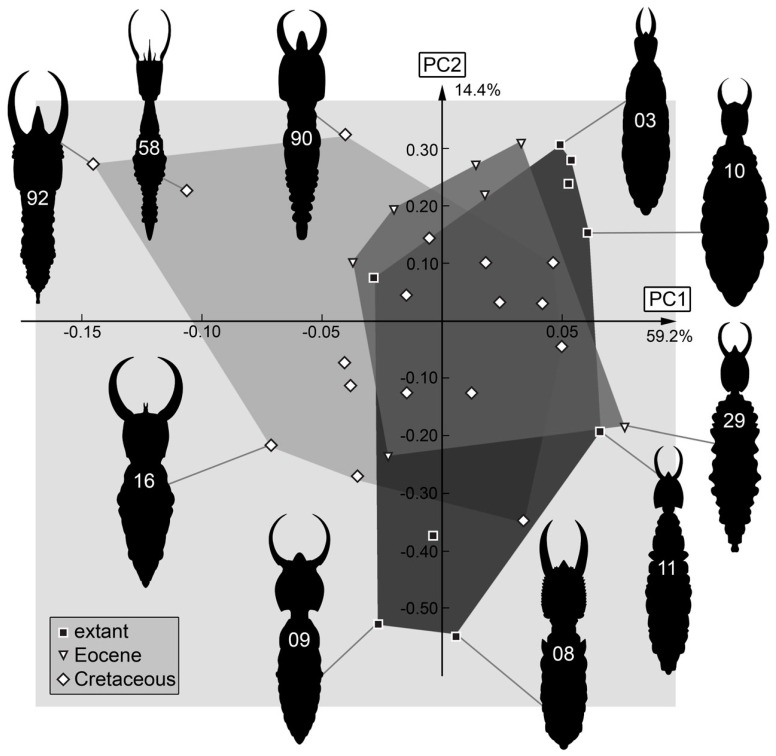
Scatterplot of PC2 vs. PC1 values of body + stylet shapes (see Supplementary Materials File S2 for details).

This dataset is smaller than those of the anterior body structures. Still, some important observations are provided. The general pattern with the Cretaceous larvae occupying the largest area is also found here (Figure 35). Yet, the size difference to the area of the modern larvae is much smaller. Moreover, the modern larvae (and some Eocene larvae) occupy areas where no Cretaceous larvae plot. This indicates that not only were certain body shapes lost, but also new ones evolved that were not yet present in the Cretaceous. It has been previously noted that modern myrmeleontiformian larvae often appear broader, while many Cretaceous larvae appear overall slenderer [114]. In other groups of lacewings, more extreme trunk shapes were already present in the Cretaceous [116].

The morphologies lost seem not only to be slenderer; in addition, the shapes have relatively larger heads and also longer stylets. This phenomenon of more elongated structures in the Cretaceous has been noted in other lacewing lineages [117,118,119,120,121].

### 4.6. Body Shape without Stylets

**Figure 36:**
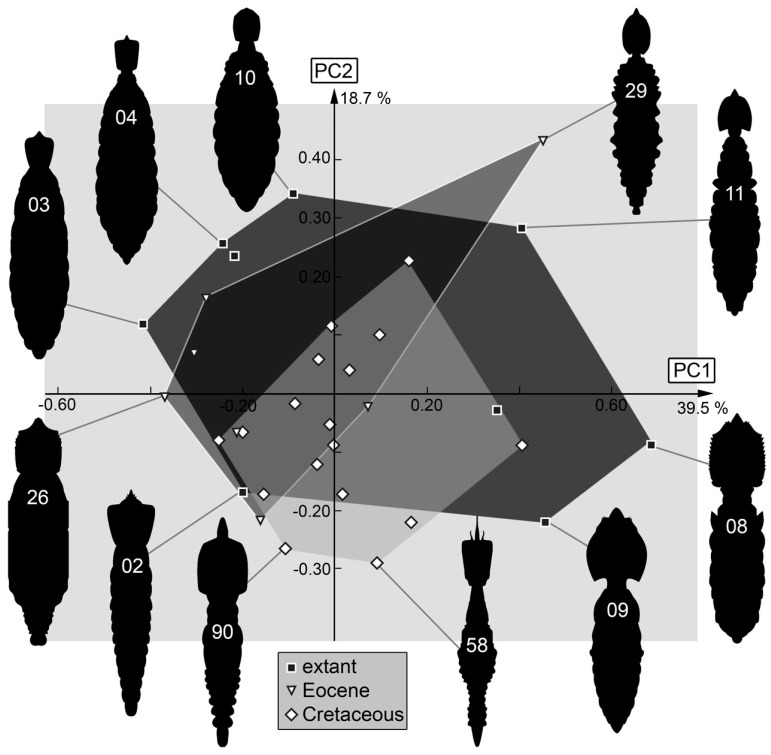
Scatterplot of PC2 vs. PC1 values of body shapes without stylets.

As we have noted regards the difference in stylets in the Cretaceous larvae, it should not be surprising that when considering the body outline without the stylets, the overall picture changes. Now, the largest occupied area is represented by the modern fauna (Figure 36). Still, the very slender bodies of some Cretaceous larvae are not represented in the modern fauna. One might argue that only three larvae plotting outside the modern morphospace may be insignificant. Yet, qualitative observations have also already hinted to the fact that myrmeleontiformian larvae had slenderer appearances in the Cretaceous [90,113,114,118]. In the light of this background, it seems likely that the difference in fossils is indeed true signal. The function of the slenderer trunk remains unclear. Yet, we need to assume that the broader trunk evolved independently several times within the modern myrmeleontiformian lineages (see discussion in [114]).

The fact that the stylets seem to play a major role in the variability in the Cretaceous larvae emphasises that the loss of morphology is likely coupled to a loss of ecological roles (see discussion in [114,119]). The differences in body shape, and the shift thereof, furthermore indicate not only a loss, but a change in ecological roles. A comparable observation was made for long-necked antlions (larvae of Crocinae [19]).

### 4.7. The Cretaceous Fauna and Its Peculiarities

As has been indicated, at a certain point, we should expect a kind of saturation effect when adding new specimens. At this point, new specimens should strongly resemble already-known ones. However, this is not yet the case.

The Cretaceous fauna seems to have a high morphological diversity due to the co-occurrence of old retained morphologies, existing very modern appearing morphologies, and aberrant experimental morphologies not known before or afterwards. Along lacewing larvae in the Cretaceous, indeed, many larvae already show very modern appearing morphologies, including indications of very modern types of behaviour [113,116,122,123,124]. Moreover, numerous highly aberrant forms are known [23,112,113,117,118,119,120,121,125,126,127,128].

Among the aberrant, now extinct forms, there are especially animals with unusual labra. Presumably, the different labrum shape also means differences in details of the feeding ecology. Moreover, the new observed morphotypes can be mostly recognised based on different labrum shapes. Unfortunately, it is still unknown how the labrum is involved in feeding for modern long-nosed antlions. In other raptorial larvae, for example, in beetles, it has been demonstrated that the shape of the labrum (and mandibles) can be directly coupled to a specialised feeding strategy (e.g., [129]). We can therefore only assume that most differences of the here-described fossil larvae and their modern counterparts are indeed related to differences in feeding strategies, but details remain unclear.

It is noteworthy that among lacewings, aberrant-appearing larvae seem to be relatively common. Among the larvae of megalopterans, the few known Cretaceous forms do not differ strongly from their modern counterparts [130]. Many beetle larvae either already appear very similar to modern larvae [131,132,133,134,135,136,137,138,139,140,141,142,143,144] or retain plesiomorphic features [134,145]. The same seems to be true for flies [146,147]; strongly aberrant experimental forms seem largely absent. Caterpillars are still very rare and do not seem to be as diversified as their modern counterparts, and without outstanding morphologies [141,148,149,150,151,152,153].

Besides lacewing larvae, among holometabolan larvae, only snakefly larvae (hence, larvae of Raphidioptera, closely related to Neuroptera) show certain peculiarities in the Cretaceous [154,155,156]. While we do not yet have quantitative data for all of these groups, it appears that the group Neuropterida, including Neuroptera, Raphidioptera, and also Megaloptera [157,158,159], had many morphologies resulting from their earlier radiations still present in the Cretaceous, but which are now extinct. This assumption is consistent with the idea that neuropteridans were part of the early radiation of Holometabola and fulfilled ecological functions nowadays performed by representatives of other groups.

### 4.8. Growth and Morphotypes

**Figure 37:**
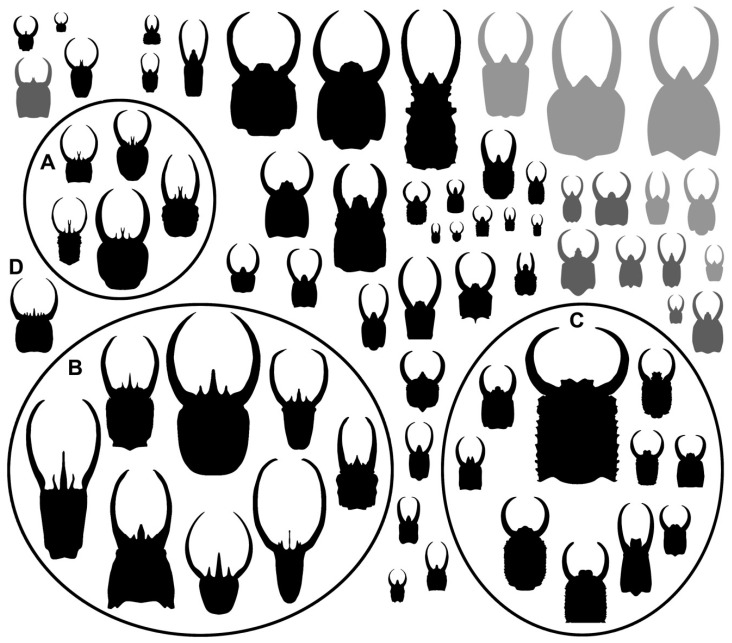
Size comparison of head + stylet shapes of all specimens for which a size indication was available, depicted at the same scale. (**A**) Trident-bearing larvae with bifurcated middle spines. (**B**) Trident-bearing larvae without a bifurcation in the middle spines. (**C**) Larvae with very broad labra. (**D**) Trident-bearing larva with additional spines.

Among other lacewing larvae, parts of the ontogenetic sequences could be recognised, providing at least first hints for recognising morphotypes or even species. So far, this has not been possible for long-nosed antlions. Haug et al. [18] speculated that larvae with bifurcated labra could always represent earlier stages. As many of these are trident-bearing larvae (Figure 37A), they should hence be smaller stages of trident-bearing larvae without a bifurcation in the middle spine (Figure 37B). Indeed, specimens with bifurcated labra are on average smaller, yet both groups show considerable variation in size, indicating that each morphotype seems to be represented by several different stages. Furthermore, there is a single specimen of the trident-bearing type possessing additional spines (in fact, a pentadent-bearing type), probably representing yet another morphotype closer related to the trident-bearing types (Figure 37D). So far, only a single species of a larva with a trident-type labrum could be formally recognised (*Acanthopsychops triaina* [113]). Yet, the large variability of the morphology clearly indicates that several species are likely represented among these larvae. Furthermore, the larvae with very broad labra (Figure 37C) likely represent not just several different stages but also several morphotypes, which again likely represent different species.

Despite the additional material, no clear ontogenetic sequences can be recognised. The main challenge in this case is that on the extant side, very little is known about the larval sequences, usually only via single-specimen reports [28,47,94,160,161,162,163,164], with the work of Tillyard [165] being the sole exception. Hence, besides more fossil material, for improving the situation, more extant specimens are required. So far, visits to collections (in Europe and Australia) have not yet provided additional specimens; hence, only aimed fieldwork may solve this issue.

## 5. Conclusions

Despite the taxonomic limitations, the analysis of fossil larvae with quantitative methods has again shown a significant loss of diversity in silky lacewings, more precisely the morphology and, coupled to this, ecology of their larvae. Although we could increase the size of the dataset significantly, we still do not see any effect of saturation, indicating that we are still seeing just a part of the original diversity back in the Cretaceous.

## Figures and Tables

**Figure 9:**
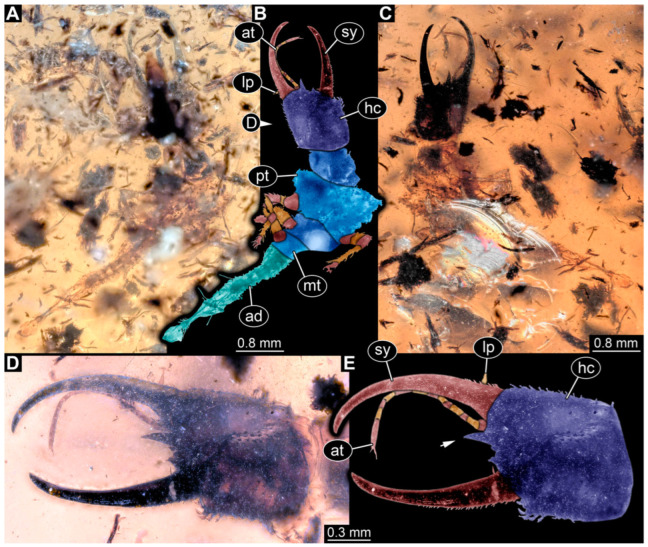
Specimen 66 (PED 0621); Myanmar amber. (**A**) Dorsal view. (**B**) Dorsal view, colour-marked. (**C**) Ventral view. (**D**) Close-up of head capsule in ventral view. (**E**) Close-up of head capsule in ventral view, colour-marked; arrow marks middle spine-like protrusion. Abbreviations: ad = abdomen; at = antenna; hc = head capsule; lp = labial palp; mt = metathorax; pt = prothorax; sy = stylet.

**Figure 10:**
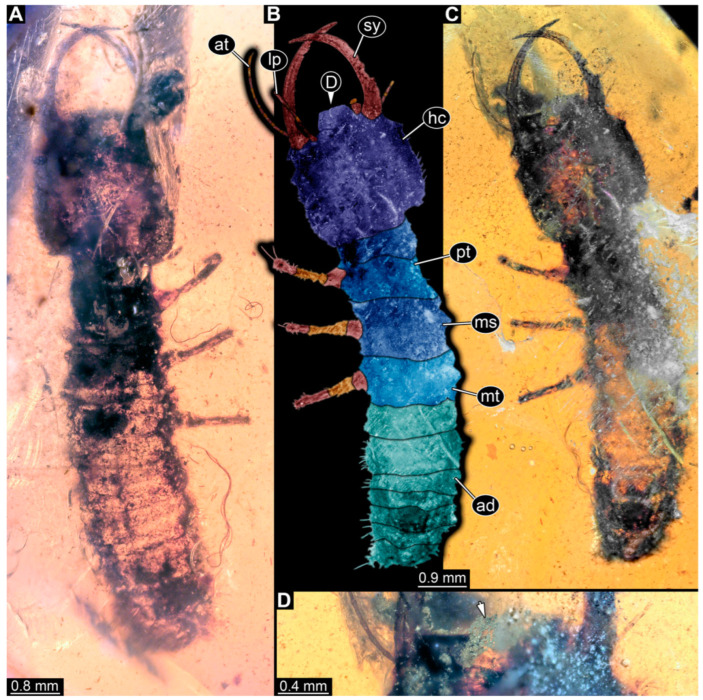
Specimen 68 (PED 0662); Myanmar amber. (**A**) Dorsal view. (**B**) Ventral view, colour-marked. (**C**) Ventral view. (**D**) Close-up of labrum in ventral view; arrow marks labrum. Abbreviations: ad = abdomen; at = antenna; hc = head capsule; lp = labial palp; ms = mesothorax; mt = metathorax; pt = prothorax; sy = stylet.

**Figure 11:**
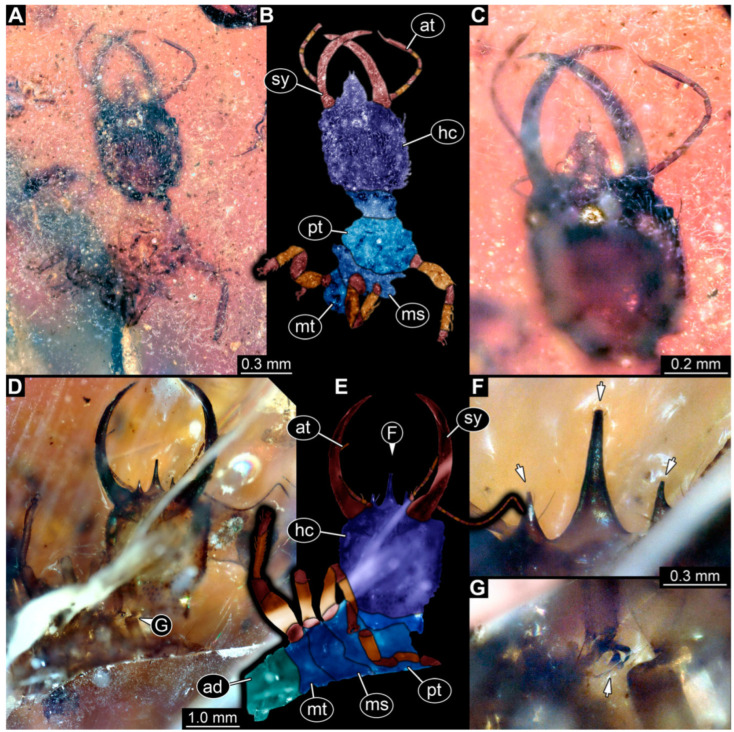
Specimens in Myanmar amber. (**A**–**C**) Specimen 70 (PED 0774). (**A**) Ventral view. (**B**) Ventral view, colour-marked. (**C**) Close-up of head capsule, ventral view. (**D**–**G**) Specimen 69 (PED 0751). (**D**) Ventral view. (**E**) Ventral view, colour-marked. (**F**) Close-up of labrum, ventral view; arrows mark spine-like protrusions. (**G**) Close-up of first locomotory appendage; arrow marks empodium. Abbreviations: ad = abdomen; at = antenna; hc = head capsule; ms = mesothorax; mt = metathorax; pt = prothorax; sy = stylet.

**Figure 12:**
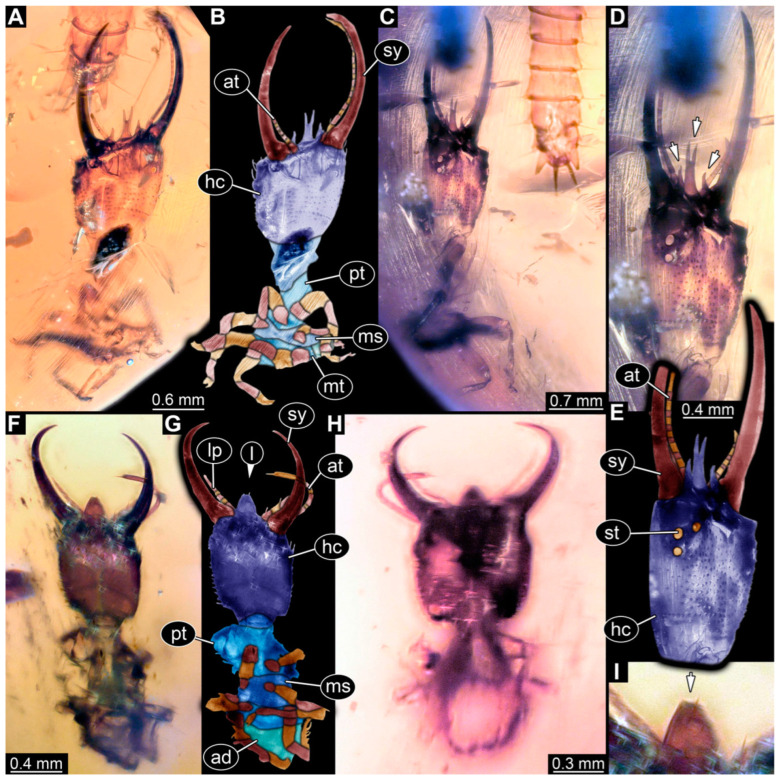
Specimens in Myanmar amber. (**A**–**E**) Specimen 72 (PED 0932). (**A**) Ventral view. (**B**) Ventral view, colour-marked. (**C**) Dorsal view. (**D**) Close-up of head capsule in dorsal view; arrows mark spine-like protrusions. (**E**) Close-up of head capsule in dorsal view, colour-marked. (**F**–**I**) Specimen 75 (PED 1459). (**F**) Ventral view. (**G**) Ventral view, colour-marked. (**H**) Dorsal view. (**I**) Close-up of labrum; arrow marks labrum. Abbreviations: ad = abdomen; at = antenna; hc = head capsule; lp = labial palp; ms = mesothorax; mt = metathorax; pt = prothorax; st = stemmata; sy = stylet.

**Figure 13:**
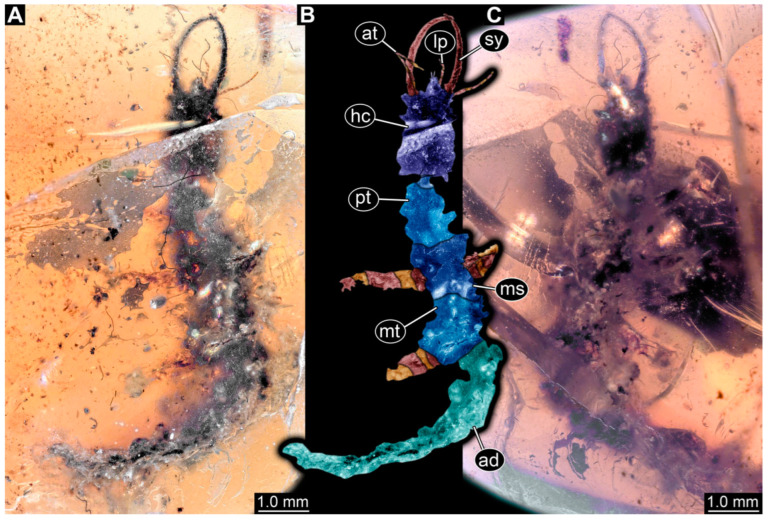
Specimen 73 (PED 0998); Myanmar amber. (**A**) Dorsal view. (**B**) Dorsal view, colour-marked. (**C**) Ventral view. Abbreviations: ad = abdomen; at = antenna; hc = head capsule; lp = labial palp; ms = mesothorax; mt = metathorax; pt = prothorax; sy = stylet.

**Figure 14:**
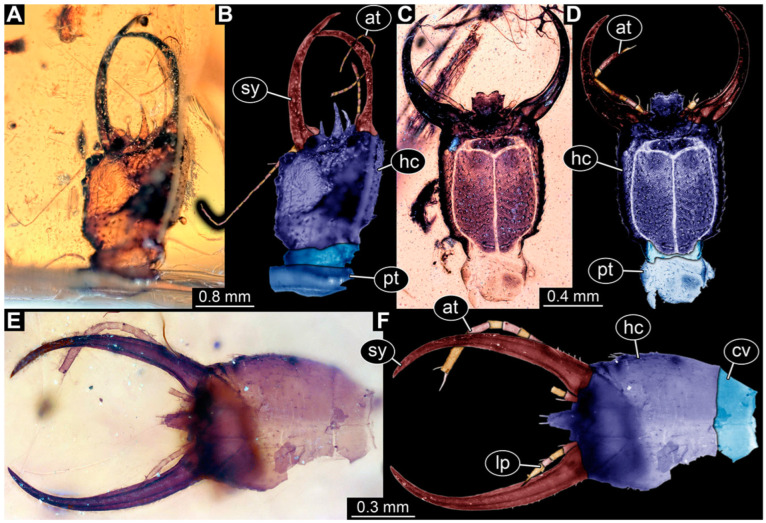
Specimens in Myanmar amber. (**A**,**B**) Specimen 74 (PED 1049). (**A**) Head capsule, dorsal view. (**B**) Head capsule, dorsal view, colour-marked. (**C**,**D**) Specimen 83 (PED 1846). (**C**) Head capsule, ventral view. (**D**) Head capsule, ventral view, colour-marked. (**E**,**F**) Specimen 82 (PED 1831). (**E**) Head capsule, ventral view. (**F**) Head capsule, ventral view, colour-marked. Abbreviations: at = antenna; hc = head capsule; lp = labial palp; pt = prothorax; sy = stylet.

**Figure 15:**
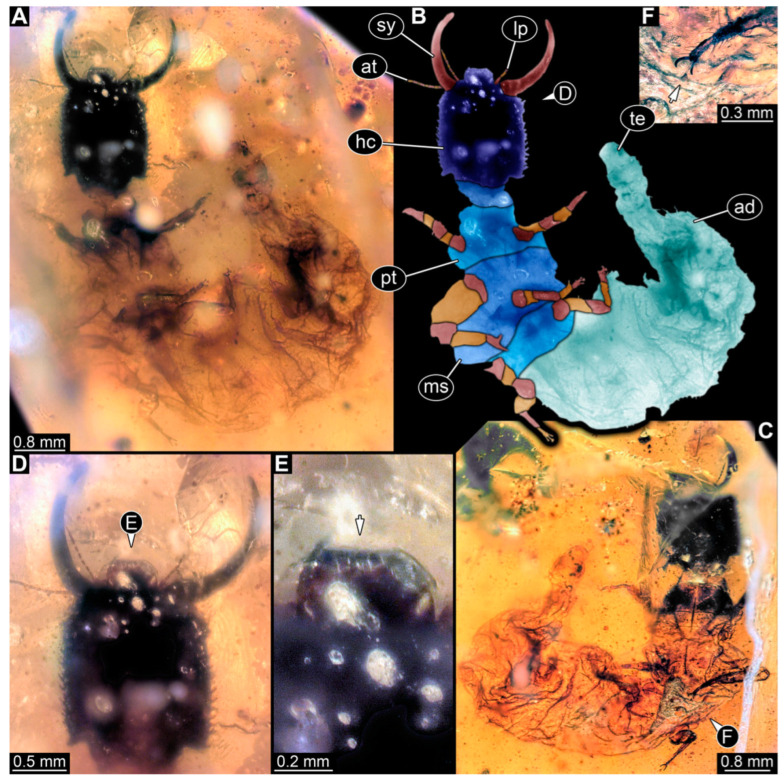
Specimen 76 (PED 1627); Myanmar amber. (**A**) Dorsal view. (**B**) Dorsal view, colour-marked. (**C**) Ventral view. (**D**) Close-up of head capsule, dorsal view. (**E**) Close-up of labrum, dorsal view; arrow marks labrum. (**F**) Close-up of second locomotory appendage; arrow marks empodium. Abbreviations: ad = abdomen; at = antenna; hc = head capsule; lp = labial palp; ms = mesothorax; mt = metathorax; pt = prothorax; sy = stylet.

**Figure 16:**
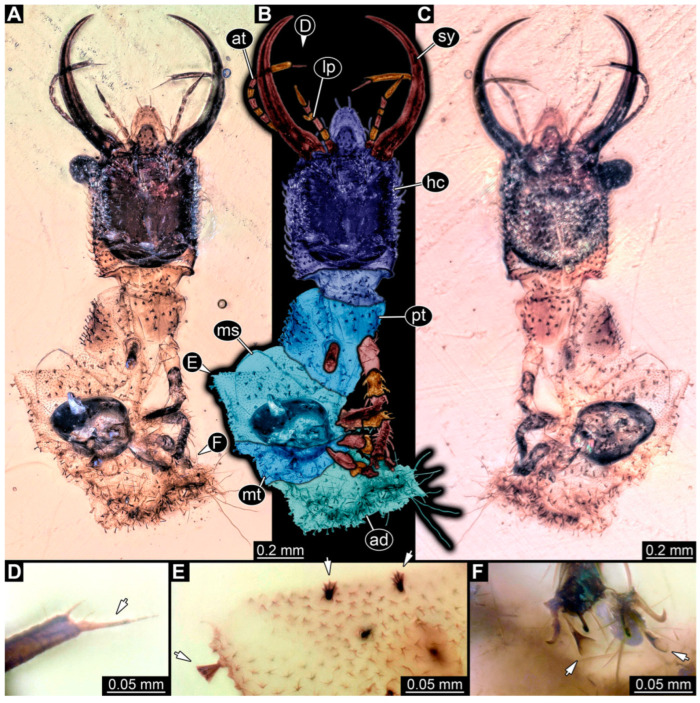
Specimen 77 (PED 1666); Myanmar amber. (**A**) Dorsal view. (**B**) Dorsal view, colour-marked. (**C**) Ventral view. (**D**) Close-up of antenna with distal seta; arrow marks seta. (**E**) Close-up of dolichasterine setae; arrows mark setae. (**F**) Close-up of second and third locomotory appendages; arrows mark empodia. Abbreviations: ad = abdomen; at = antenna; hc = head capsule; lp = labial palp; ms = mesothorax; mt = metathorax; pt = prothorax; sy = stylet.

**Figure 17:**
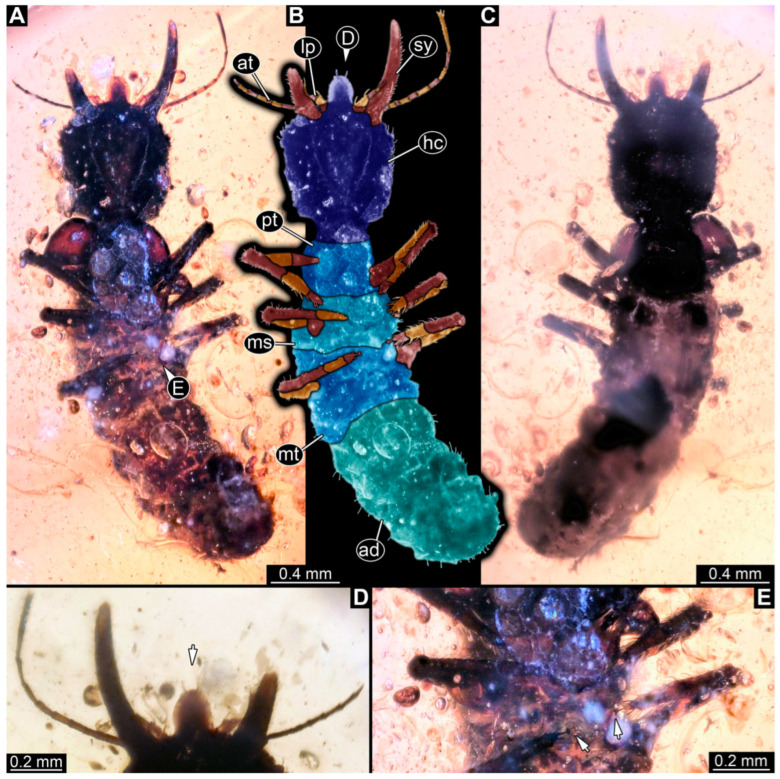
Specimen 78 (PED 1703); Myanmar amber. (**A**) Ventral view. (**B**) Ventral view, colour-marked. (**C**) Dorsal view. (**D**) Close-up of labrum in dorsal view; arrow marks labrum. (**E**) Close-up of locomotory appendages; arrows mark empodia. Abbreviations: ad = abdomen; at = antenna; hc = head capsule; lp = labial palp; ms = mesothorax; mt = metathorax; pt = prothorax; sy = stylet.

**Figure 18:**
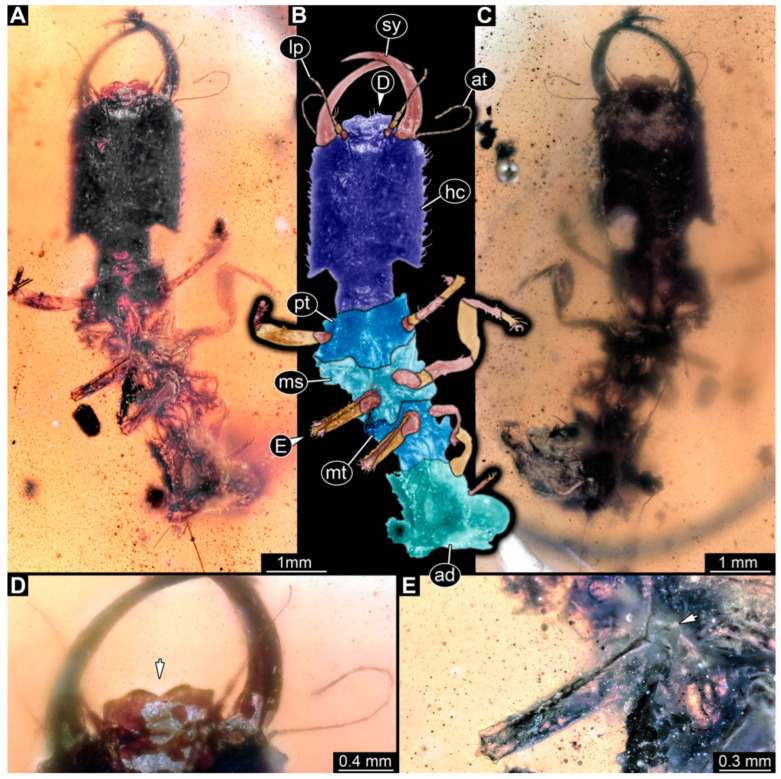
Specimen 79 (PED 1726); Myanmar amber. (**A**) Ventral view. (**B**) Ventral view, colour-marked. (**C**) Dorsal view. (**D**) Close-up of labrum in ventral view; arrow marks a broad cleft in the labrum. (**E**) Close-up of second locomotory appendage; arrow marks empodium. Abbreviations: ad = abdomen; at = antenna; hc = head capsule; lp = labial palp; ms = mesothorax; mt = metathorax; pt = prothorax; sy = stylet.

**Figure 19:**
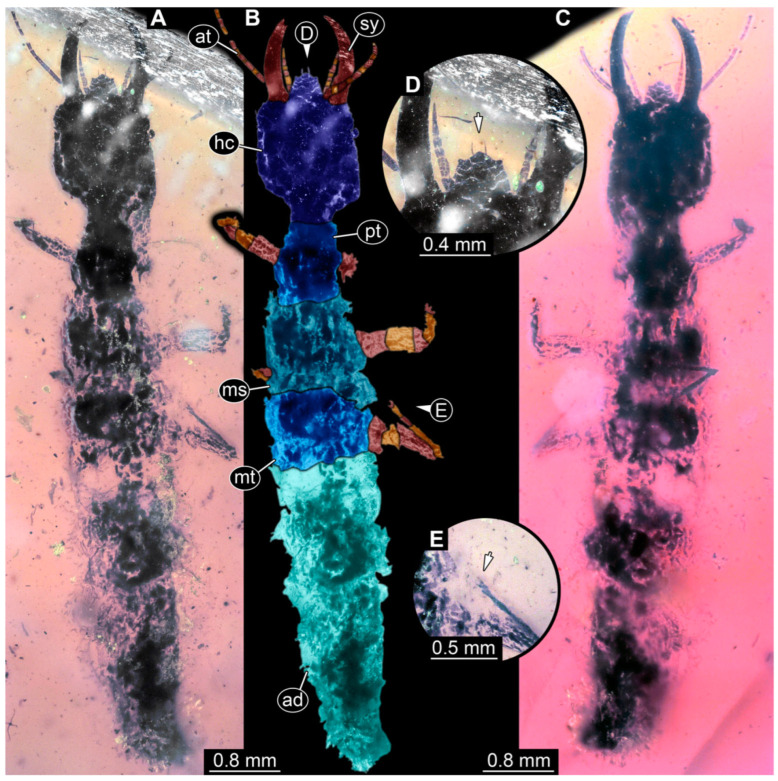
Specimen 80 (PED 1732); Myanmar amber. (**A**) Dorsal view. (**B**) Dorsal view, colour-marked. (**C**) Ventral view. (**D**) Close-up of labrum in dorsal view; arrow marks the labrum. (**E**) Close-up of third locomotory appendage; arrow marks distal end. Abbreviations: ad = abdomen; at = antenna; hc = head capsule; ms = mesothorax; mt = metathorax; pt = prothorax; sy = stylet.

**Figure 20:**
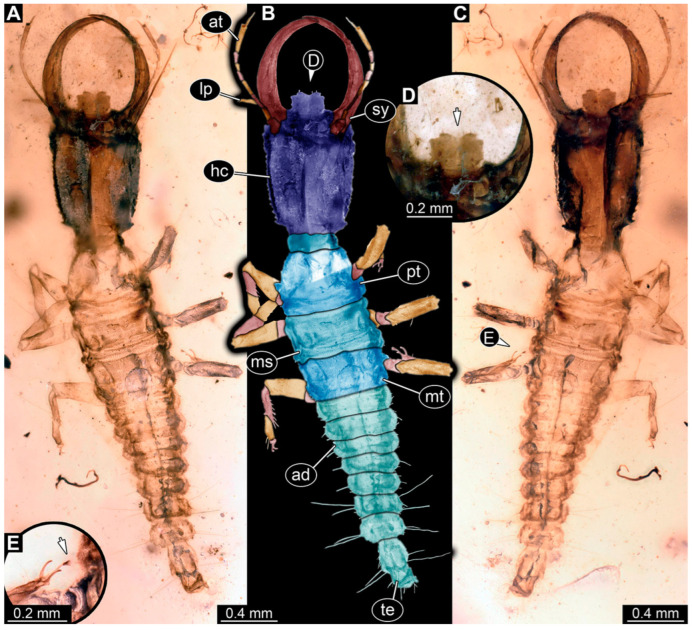
Specimen 81 (PED 1813); Myanmar amber. (**A**) Dorsal view. (**B**) Dorsal view, colour-marked. (**C**) Ventral view. (**D**) Close-up of labrum in dorsal view; arrow marks middle cleft of the labrum. (**E**) Close-up of third locomotory appendage; arrow marks empodium. Abbreviations: ad = abdomen; at = antenna; hc = head capsule; lp = labial palp; ms = mesothorax; mt = metathorax; pt = prothorax; sy = stylet; te = trunk end.

**Figure 21:**
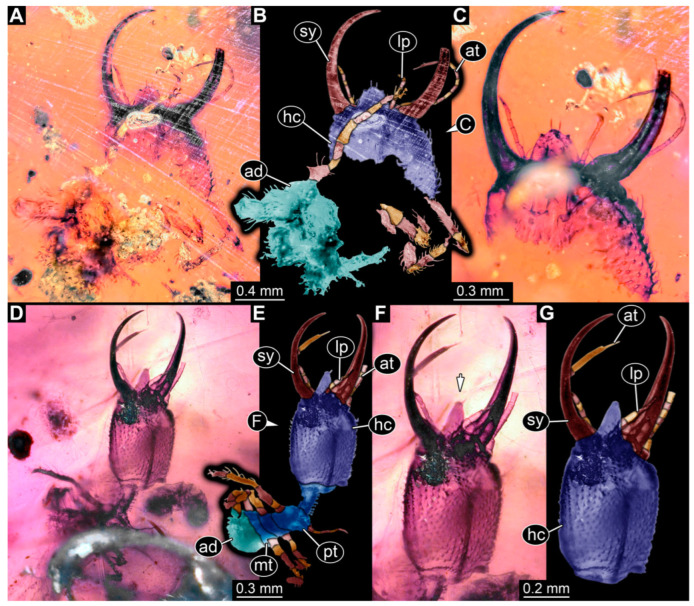
Specimens in Myanmar amber. (**A**–**C**) Specimen 85 (PED 1887). (**A**) Ventral view. (**B**) Ventral view, colour-marked. (**C**) Close-up of labrum in ventral view. (**D**–**G**) Specimen 87 (PED 1940). (**D**) Dorsal view. (**E**) Dorsal view, colour-marked. (**F**) Close-up of head; arrow marks labrum. (**G**) Close-up of head, colour-marked. Abbreviations: ad = abdomen; at = antenna; hc = head capsule; lp = labial palp; mt = metathorax; pt = prothorax; sy = stylet.

**Figure 22:**
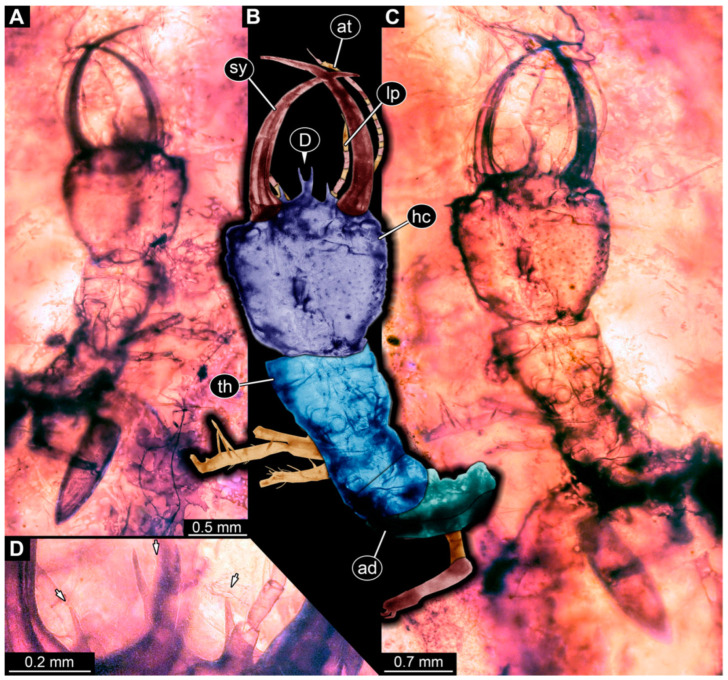
Specimen 86 (PED 1928); Myanmar amber. (**A**) Ventral view. (**B**) Dorsal view, colour-marked. (**C**) Dorsal view. (**D**) Close-up of labrum in dorsal view; arrows mark spine-like protrusions. Abbreviations: ad = abdomen; at = antenna; hc = head capsule; lp = labial palp; sy = stylet; th = thorax.

**Figure 23:**
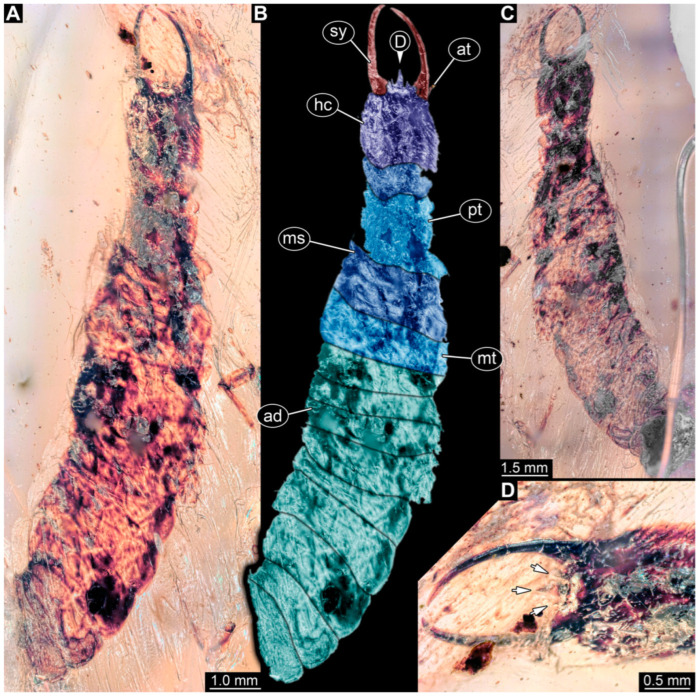
Specimen 88 (PED 1967); Myanmar amber. (**A**) Dorsal view. (**B**) Dorsal view, colour-marked. (**C**) Ventral view. (**D**) Close-up of head capsule in ventral view; arrows mark spine-like protrusions of the labrum. Abbreviations: ad = abdomen; at = antenna; hc = head capsule; ms = mesothorax; mt = metathorax; pt = prothorax; sy = stylet.

**Figure 24:**
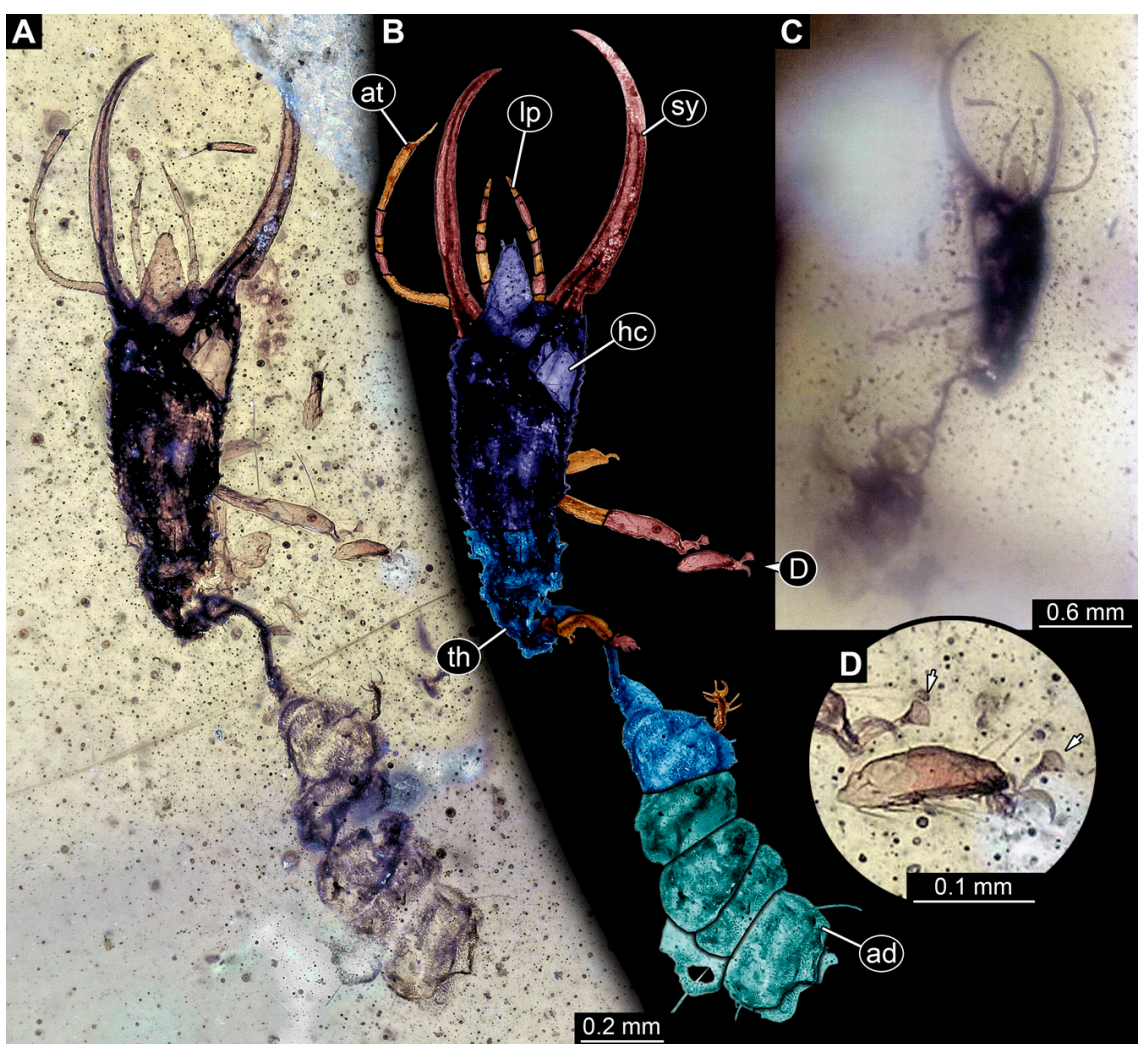
Specimen 89 (PED 2056); Myanmar amber. (**A**) Dorsal view. (**B**) Dorsal view, colour-marked. (**C**) Ventral view. (**D**) Close-up of locomotory appendages; arrows mark empodia. Abbreviations: ad = abdomen; at = antenna; hc = head capsule; lp = labial palp; sy = stylet; th = thorax.

**Figure 25:**
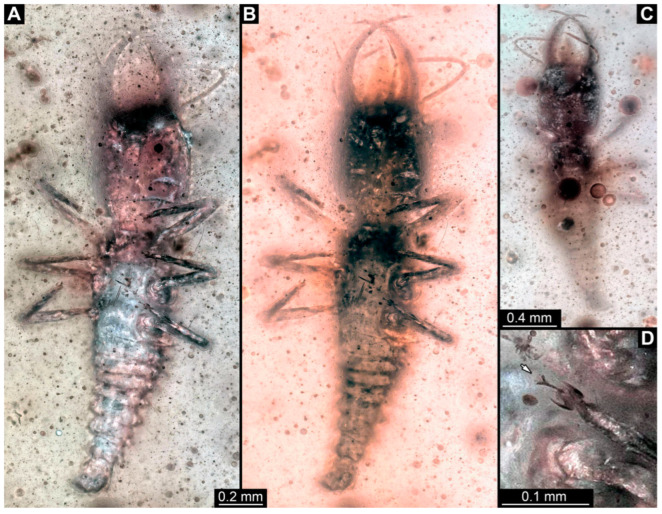
Specimen 90 (PED 2171); Myanmar amber. (**A**,**B**) Ventral view under different illuminations. (**C**) Dorsal view. (**D**) Close-up of locomotory appendages; arrow marks empodium.

**Figure 26:**
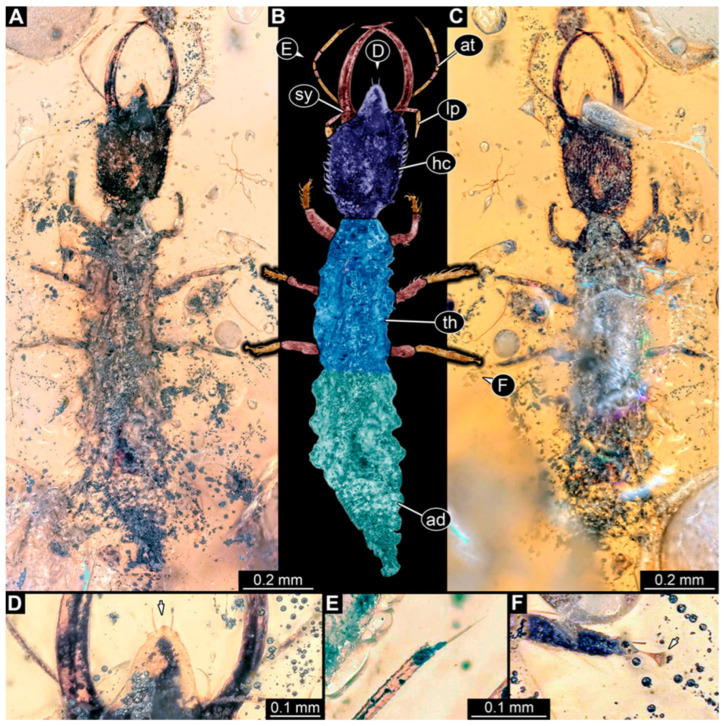
Specimen 91 (PED 2309); Myanmar amber. (**A**) Ventral view. (**B**) Ventral view, colour-marked. (**C**) Dorsal view. (**D**) Close-up of labrum; arrow marks labrum. (**E**) Close-up of antenna. (**F**) Close-up of third locomotory appendage; arrow marks empodium. Abbreviations: ad = abdomen; at = antenna; hc = head capsule; lp = labial palp; sy = stylet; th = thorax.

**Figure 27:**
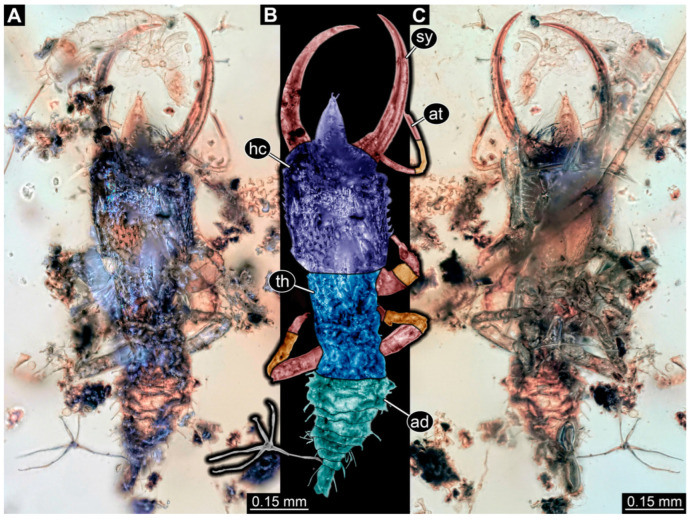
Specimen 92 (PED 2311); Myanmar amber. (**A**) Dorsal view. (**B**) Dorsal view, colour-marked. (**C**) Ventral view. Abbreviations: ad = abdomen; at = antenna; hc = head capsule; sy = stylet; th = thorax.

**Figure 28:**
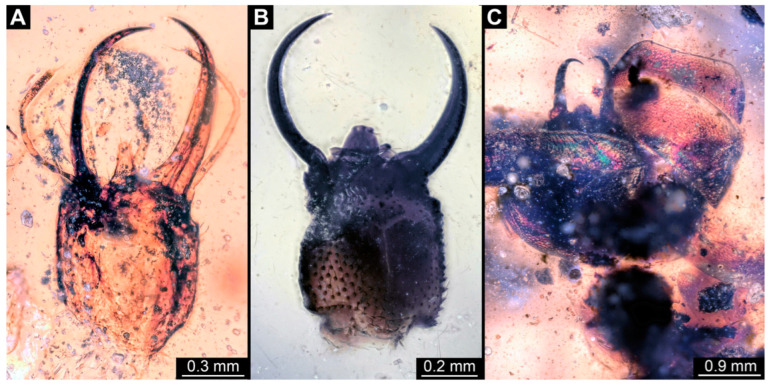
Specimens in Myanmar amber. (**A**) Specimen 94 (PED 2446). (**B**) Specimen 93 (PED 2329). (**C**) Specimen 95 (PED 2448).

**Figure 29:**
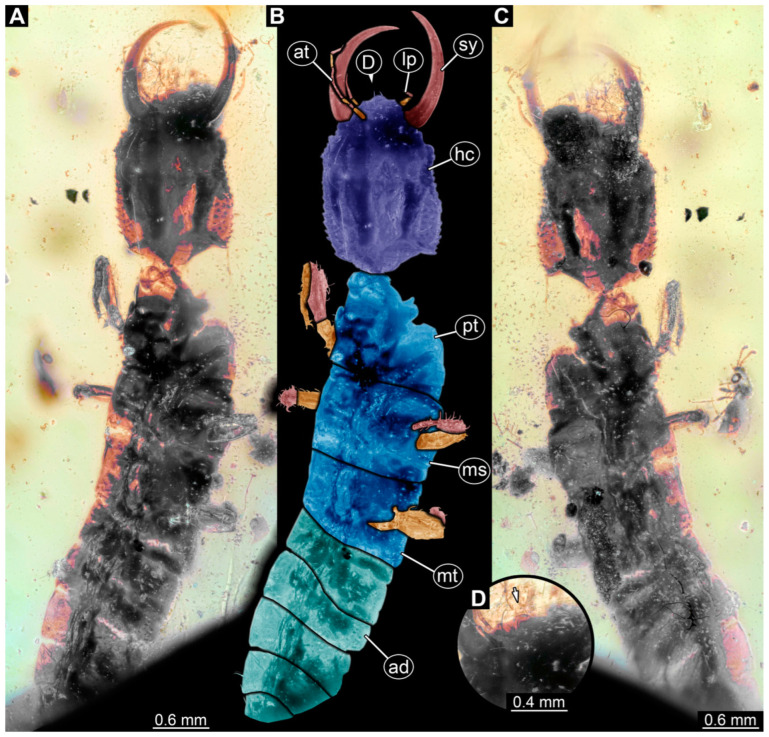
Specimen 96 (PED 2432); Myanmar amber. (**A**) Ventral view. (**B**) Ventral view, colour-marked. (**C**) Dorsal view. (**D**) Close-up of labrum; arrow marks labrum. Abbreviations: ad = abdomen; at = antenna; hc = head capsule; lp = labial palp; ms = mesothorax; mt = metathorax; pt = prothorax; sy = stylet.

**Figure 30:**
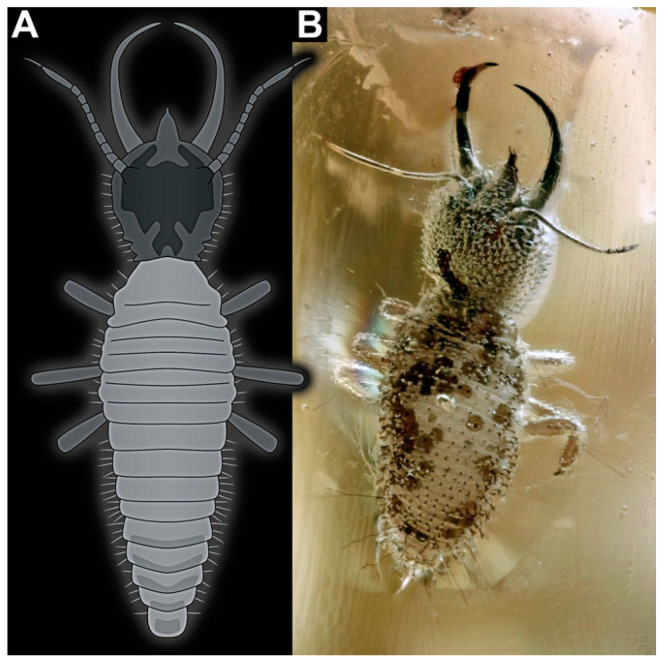
Specimens in Baltic amber; not to scale. (**A**) Specimen 97, modified after Ross [104]. (**B**) Specimen 98, provided by Marius Veta (www.ambertreasure4u.com, accessed on 23 December 2022).

## Data Availability

All data from this study are available in this paper and associated papers. Figure 1, Figure 2, Figure 3, Figure 4, Figure 5, Figure 6, Figure 7, Figure 8, Figure 9, Figure 10, Figure 11, Figure 12, Figure 13, Figure 14, Figure 15, Figure 16, Figure 17, Figure 18, Figure 19, Figure 20, Figure 21, Figure 22, Figure 23, Figure 24, Figure 25, Figure 26, Figure 27, Figure 28, Figure 29, Figure 30, Figure 31, Figure 32, Figure 33, Figure 34, Figure 35, Figure 36 and Figure 37 of high resolution are available at https://doi.org/10.5281/zenodo.7585157 (accessed on 23 December 2022).

## Supplementary Materials

The following supporting information can be downloaded at: https://www.mdpi.com/article/10.3390/insects14020170/s1, File S1. Information on the specimens included in the analyses. Abbreviations: Eoc = Eocene; K = Cretaceous; Mio = Miocene. File S2. Files resulting from the shape analyses.
