# Supplementary figures and images for: An Expanded View on the Morphological Diversity of Long-Nosed Antlion Larvae Further Supports a Decline of Silky Lacewings in the Past 100 Million Years
Source: Insects. 2023 Feb 9;14(2):170. doi: 10.3390/insects14020170 (PMC9966087; doi:10.3390/insects14020170)

-2S.D.

Mean

+2S.D.

PC1

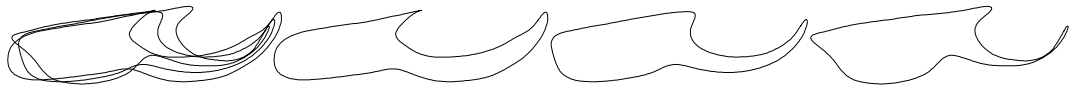

PC2

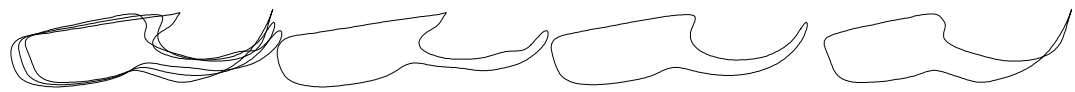

PC3

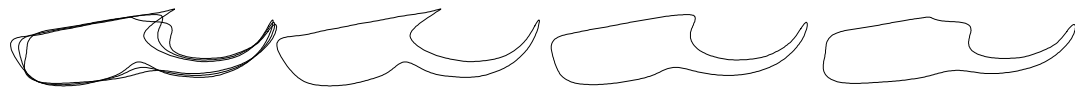

PC4

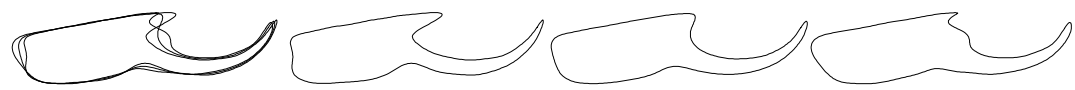

PC5

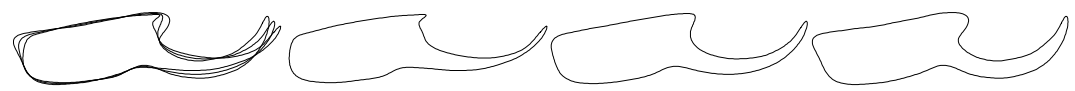

-2S.D.

Mean

+2S.D.

PC6

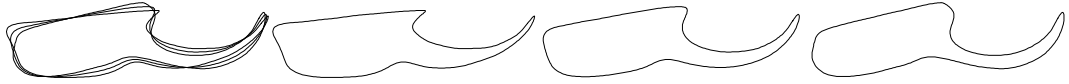

Supplement: Supplementary file 1 [file insects-14-00170-s001.zip › SupplFilesPsycho2/SupplFileS2_Psycho2/01_head_n_stylet.pdf]

-2S.D.

Mean

+2S.D.

PC1

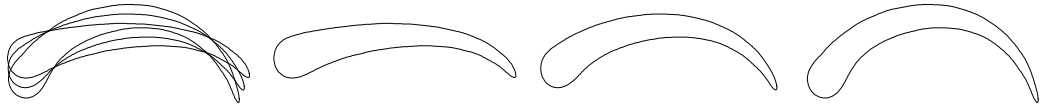

PC2

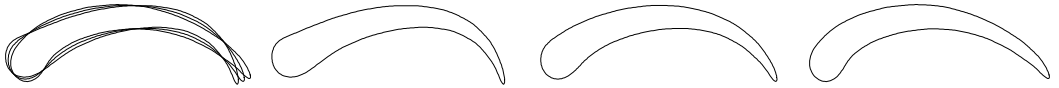

PC3

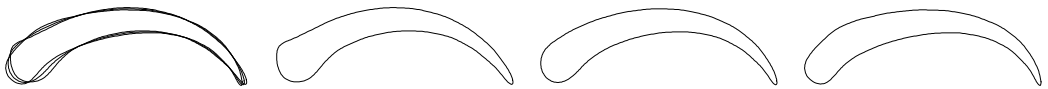

PC4

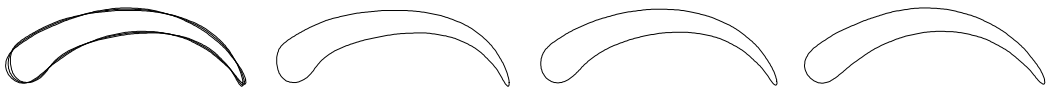

Supplement: Supplementary file 1 [file insects-14-00170-s001.zip › SupplFilesPsycho2/SupplFileS2_Psycho2/02_stylet.pdf]

-2S.D.

Mean

+2S.D.

PC1

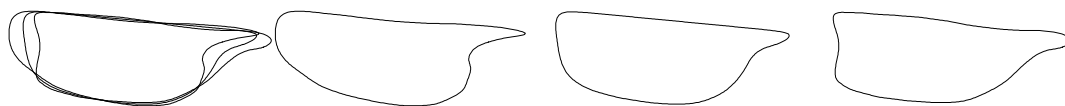

PC2

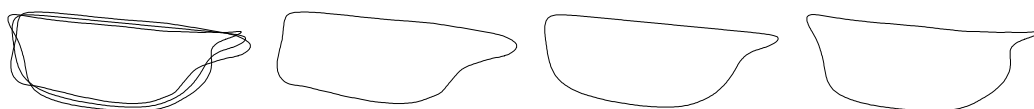

PC3

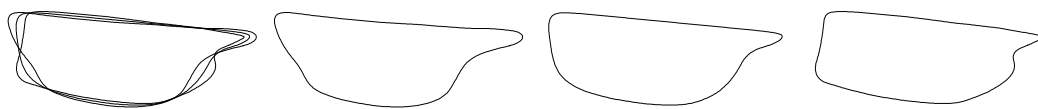

PC4

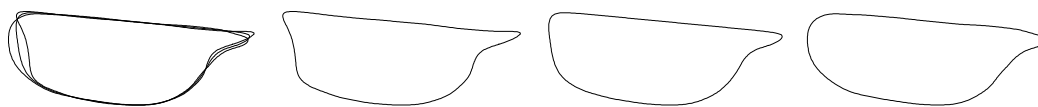

PC5

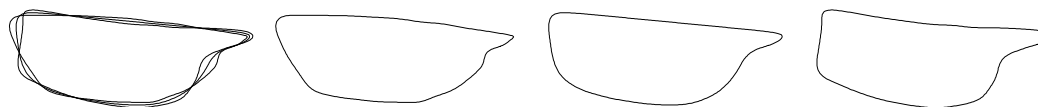

-2S.D.

Mean

+2S.D.

PC6

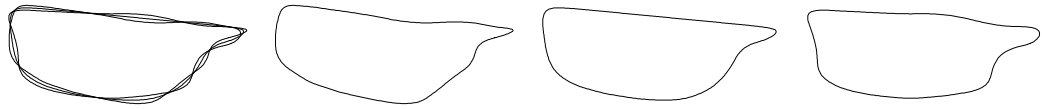

PC7

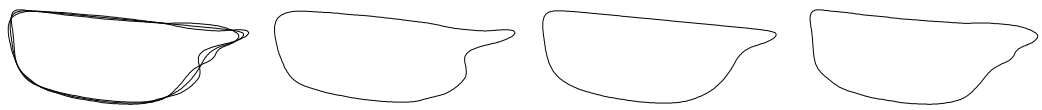

PC8

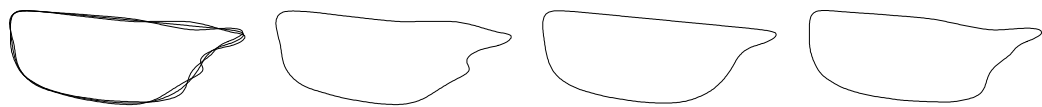

Supplement: Supplementary file 1 [file insects-14-00170-s001.zip › SupplFilesPsycho2/SupplFileS2_Psycho2/03_head_only.pdf]

-2S.D.

Mean

+2S.D.

PC1

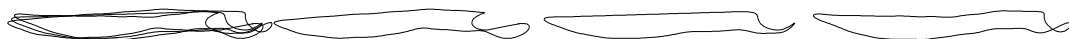

PC2

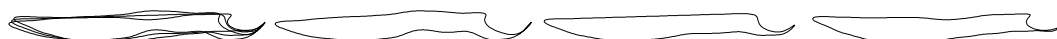

PC3

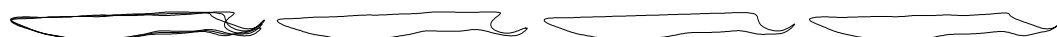

PC4

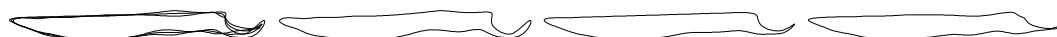

PC5

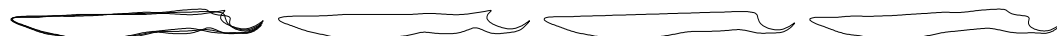

-2S.D.

Mean

+2S.D.

PC6

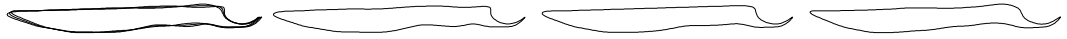

Supplement: Supplementary file 1 [file insects-14-00170-s001.zip › SupplFilesPsycho2/SupplFileS2_Psycho2/04_body_w_stylet.pdf]

-2S.D.

Mean

+2S.D.

PC1

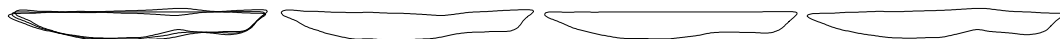

PC2

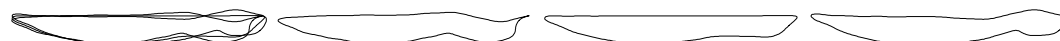

PC3

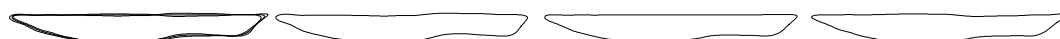

PC4

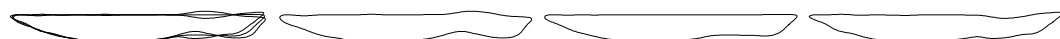

PC5

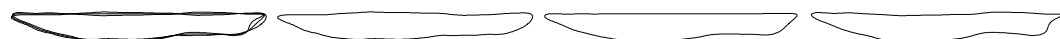

-2S.D.

Mean

+2S.D.

PC6

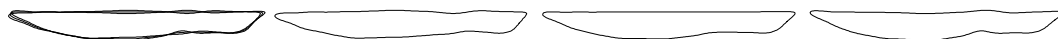

PC7

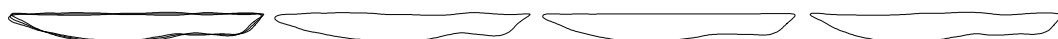

PC8

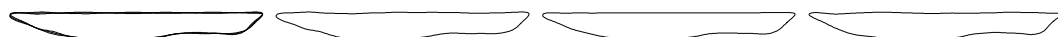

Supplement: Supplementary file 1 [file insects-14-00170-s001.zip › SupplFilesPsycho2/SupplFileS2_Psycho2/05_body_wo_stylet.pdf]
